# Tigers in the Neighborhood: Factors Associated With Fatal Tiger Attacks in a Human‐Dominated Landscape, Nepal

**DOI:** 10.1002/ece3.74206

**Published:** 2026-08-16

**Authors:** Bed Kumar Dhakal, Babu Ram Lamichhane, Sanjay Kandel, Naresh Subedi, Matthew Low, Thakur Silwal

**Affiliations:** ^1^ Tribhuvan University, Institute of Forestry Kathmandu Nepal; ^2^ Department of National Parks and Wildlife Conservation Kathmandu Nepal; ^3^ Wild Care Nepal Lalitpur Nepal; ^4^ ZSL Nepal Kathmandu Nepal; ^5^ National Trust for Nature Conservation Lalitpur Nepal; ^6^ Department of Ecology Swedish University of Agricultural Sciences Uppsala Sweden

**Keywords:** buffer zone, co‐existence, human death and injury, human‐tiger‐conflict, Terai Arc Landscape

## Abstract

Effective conservation of tigers ( *Panthera tigris* ) in human‐dominated landscapes requires dedicated efforts for careful management of human‐tiger conflict. Tiger attacks on humans are the extreme form of conflict occurring primarily during livelihood activities in the Terai Arc Landscape of Nepal. We assessed spatio‐temporal patterns of tiger attacks on humans and factors associated with human fatality. Data of tiger attacks on humans were collected from registered cases of national park offices and division forest offices. We interviewed 245 victims or family members attacked by tigers between 2010 and 2024 using a semi‐structured questionnaire. Most tiger attacks on humans (> 90%) occurred inside forest areas and over half were fatal. Men were attacked more frequently (65.3%) but fatality was higher among women (> 70%). The majority of the victims (93%) had education of secondary level or below. The frequency of tiger attacks on people of different ethnic groups was proportionate to their population. A large number of the tiger attacks occurred during forest resource collection or livestock herding. The victims' group size, sex, education level and posture during the tiger attacks affected human fatality. Tiger attacks can be minimized by applying adaptive management of buffer zone and corridor forests, adopting safety protocols through behavior change campaigns, promoting alternative livelihoods and tiger tourism, and proactive management of conflict‐causing tigers. The findings of this research have long‐term implications to strengthen human‐tiger co‐existence in human‐dominated landscapes in Nepal and similar landscapes.

## Graphical abstract

We documented 245 tiger attacks on humans (141 fatalities) during 2010–2024 in Nepal. Most of the attacks (> 90%) occurred inside forest areas. Men were attacked more frequently (65.3%) but fatality was higher among women (> 70%). The majority of the victims (93%) had education of secondary level or below.

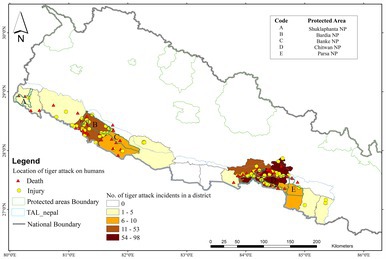

## 1 Introduction

Conserving tigers ( *Panthera tigris* ) in human‐dominated landscapes is fundamentally a social–ecological challenge and needs careful management of social and ecological conditions necessary for coexistence (Redpath et al. 2013; Struebig et al. 2018; Jhala et al. 2025). Tigers need large and intact natural habitats with adequate prey species (Smith et al. 1998; Inskip and Zimmermann 2009), yet habitat loss and fragmentation (Acharya et al. 2017), prey depletion (Karanth et al. 2004), unplanned human settlements in proximity to forests (Wegge et al. 2018), and infrastructure developments and climate change (Seidensticker et al. 2010) are increasingly bringing tiger populations and local communities into close contact (Inskip and Zimmermann 2009). The resulting human tiger conflict (HTC) imposes severe costs on both tigers and people. For tigers, threat of poisoning and retaliatory killings increases, which have adverse effects on tiger populations. Human‐caused mortality is a major threat to tigers (Chapron et al. 2008, 2014; Goodrich 2010; Sanderson et al. 2019).

Similarly, for people sharing landscapes with tigers, livestock depredation threatens local livelihoods (Lamichhane et al. 2018; Miller 2020), and the risk of attack leading to injury or death develops the fear, insecurity and stigma in affected communities (Gurung et al. 2008). If such incidents increased, it could reduce the support and local legitimacy on which sustainable tiger conservation increasingly depends (Nyhus 2016; Rastogi et al. 2022). Furthermore, the costs of HTC are not borne evenly within societies, but women and forest‐dependent communities bear disproportionate physical, economic and psychosocial costs (Ogra 2008; Doubleday and Adams 2020; Stevens et al. 2025; Adler et al. 2026).

Visible costs of HTC including deaths, injuries and livestock loss are countable and relief support is provided for this loss, while its hidden costs remain uncompensated. Moreover, the psychosocial burdens such as fear, stigma, and long‐term psychosocial and opportunity costs for forest‐dependent households fall disproportionately and are under‐recognized (Barua et al. 2013; Chowdhurym et al. 2016; Sharma et al. 2021).

Managing HTC is one of the major challenges for most of the tiger‐range countries where both the human and tiger populations are simultaneously increasing (Wikramanayake et al. 2010). With an increasing tiger population and landscape use, the probability of human tiger confrontation in the form of tiger attacks on human, livestock depredation, and retaliatory killing of tigers by affected communities increases (Struebig et al. 2018). The survival of tigers will ultimately depend upon increasing the tolerance level of the communities living alongside them (Goodrich 2010), which in turn, is determined by how well conflict is managed, and ownership taken by the communities (Jhala et al. 2025).

In developed countries rising attacks are often linked to recreational outdoor activity (Penteriani et al. 2016), whereas in developing countries attacks remain overwhelmingly linked to routine forest‐based livelihood activities, particularly the collection of fuelwoods, fodder, and NTFPs (Silwal et al. 2017; Bombieri et al. 2023). Moreover, studies from developing countries, especially from South Asia have documented how cultural norms, livelihood dependencies and local institutions shape both the costs and conservation outcomes of human‐tiger coexistence (Rastogi et al. 2014; Aiyadurai 2016; Stevens et al. 2025).

Tiger attacks on humans are extreme form of conflict (Dhanwatey et al. 2013). Tigers might attack people as prey, or defensively to protect themselves (and their cubs) particularly when persecuted by people (Gurung et al. 2008; Goodrich 2010). To reduce tiger attacks on humans requires information on where and when encounters occur, and the human and ecological attributes associated with such incidents (Dhanwatey et al. 2013; Dhar and Mondal 2023). These dimensions should be interpreted jointly because spatial overlap creates encounter possibilities, whereas social and situational conditions shape vulnerability and the consequences of attacks (Dhanwatey et al. 2013). Thus, understanding the dynamics of tiger attacks on humans is critical not only for tiger conservation but also for the welfare of people living in the shared landscape. A comprehensive understanding of spatial, temporal, behavioral and demographic patterns of tiger attacks on humans is necessary to develop effective conservation strategies (Goodrich 2010).

Nepal is typical example of the tiger range countries which is facing the intense human tiger conflict. As a signatory country of global tiger recovery program, Nepal committed to double its tiger population by 2022 (GTRP 2011; Paudel et al. 2024) and invested substantial efforts on securing tiger population and their habitat, reducing poaching, prey recovery and managing human‐tiger conflict. As a result, tiger population nearly tripled from baseline of 121 in 2009 to 355 in 2022 (DNPWC and DFSC 2022). With significant increment of tiger population, there are also growing concerns of human‐tiger conflict (Pandey et al. 2026; Paudel et al. 2024). However, information on dynamics of human‐tiger conflict and its impacts on the forest dependent communities is limited.

Studies examining patterns of HTC, particularly, tiger attack on human in Terai Arc Landscape (TAL) are location specific (protected areas or corridors), focusing on spatial, temporal and demographic characteristics of human‐tiger encounters (Gurung et al. 2008; Bhattarai and Fischer 2014; Ramesh and Malviya 2015; Silwal et al. 2017; Dhungana et al. 2018). However, no comprehensive studies at the landscape level are available in Nepal. Here, we present insights on tiger attacks on human in Nepal's TAL for the period of 2010–2024. We assessed the socio‐economic characteristics of the victims, spatial and temporal patterns and factors associated with risk of fatality on tiger attacks. The findings of the study have long‐term implications for strengthening human tiger co‐existence in Nepal and elsewhere.

## 2 Methods

### 2.1 Study Area

**FIGURE 1:**
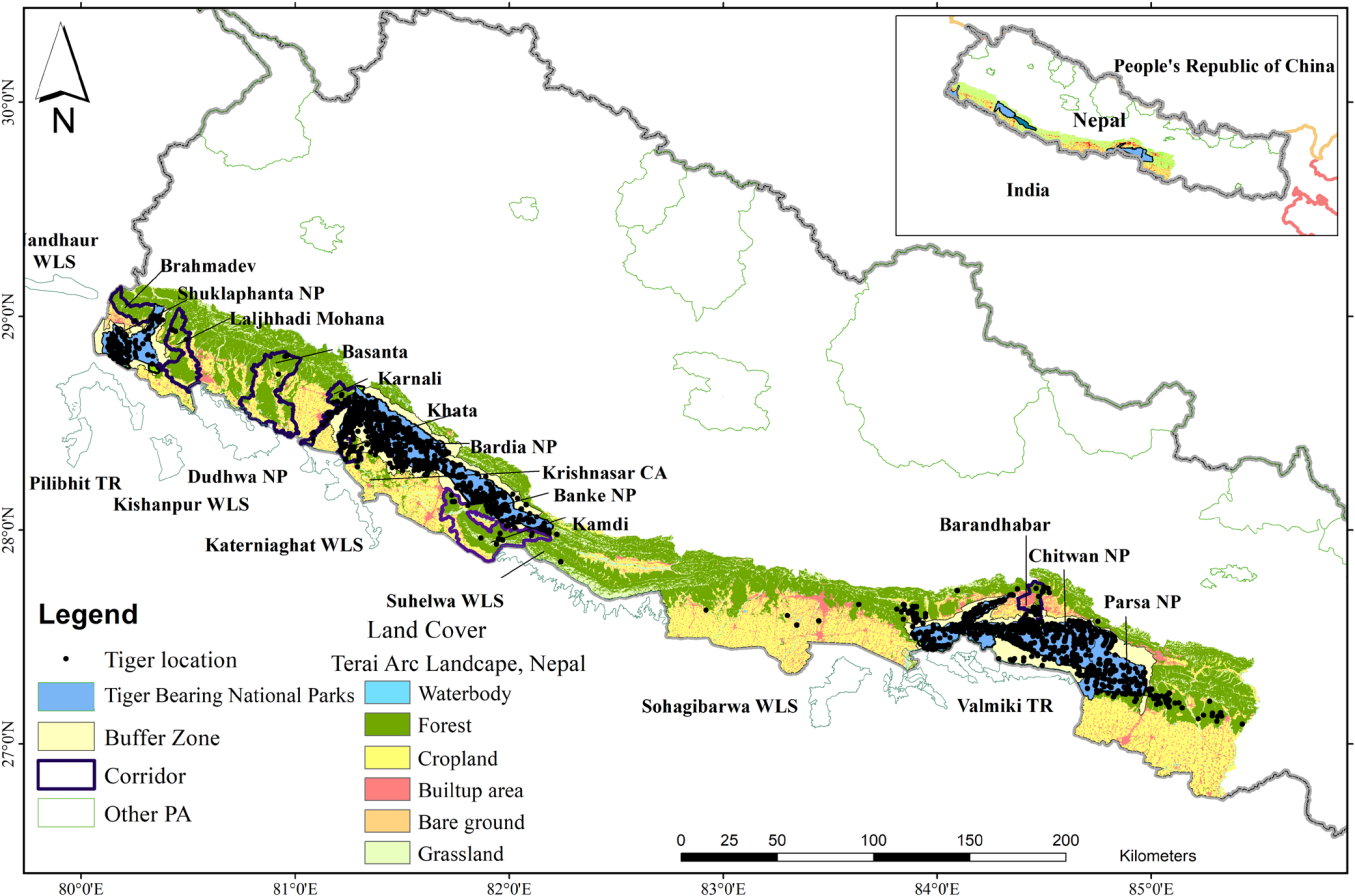
Terai Arc Landscape, Nepal with tiger presence locations, protected areas, forest corridors, and other land use types.

The study was conducted in the Nepal part of the transboundary Terai Arc Landscape (TAL) (hereinafter TAL) (Figure 1), covering an area of 24,710 km^2^ of Terai plains and Himalayan foothills. TAL is one of the high priority landscapes for global tiger conservation (MOFSC 2015) with a mosaic of protected areas, government managed forests, community forests, agricultural land and built‐up areas. Tigers occur in five national parks (NPs) (Parsa, Chitwan, Banke, Bardia and Shuklaphanta) and adjoining forests in Nepal. To create large and inviolate space for wildlife including tigers, and reduce human‐wildlife conflict, a total of 922.52 km^2^ (Pandey et al. 2026) of core protected areas was extended by community resettlement (Pandey et al. 2026) between 1973 and 2013. Most of resettlements were voluntary and resettled communities have improved living standards with continued support of the government and conservation partners (Mclean and Straede 2003; Dhakal et al. 2011). Moreover, such resettlements have contributed to recovery of tigers and many other wildlife species (Subedi et al. 2013; Lamichhane et al. 2018).

Tiger bearing protected areas of Nepal are connected by forest and riparian corridors that facilitate movement of tigers (Wikramanayake et al. 2004; Thapa and Kelly 2017; Subedi et al. 2021). Chitwan NP shares park boundaries with Valmiki Tiger Reserve (India), Banke NP is connected with Suhelwa Wildlife Sanctuary (India) via the Kamdi corridor, Bardia NP is connected to Katerniaghat Wildlife Sanctuary (India) via Khata corridor, and Shuklaphanta NP is connected with Pilibhit Tiger Reserve (India) and Dudhwa Tiger Reserve (India) via Lagga Bagga (India) and Laljhadi forest corridors respectively. These corridors and connectivity are central to maintaining ecological processes and sustaining a viable metapopulation of endangered species like tigers. Recovery of tiger population has been one of the great conservation successes in TAL (Bhatt et al. 2023).

The TAL is not only ecologically significant but also a socioeconomically complex human‐dominated landscape. TAL has heterogeneous society with mix of hill migrants (36.5%), indigenous Terai communities (17.8%) including Tharu, Bote, Kumal, Majhi, Musahar etc., Dalit (12.5%) and Others (33.2%) differing in settlement histories, traditional knowledge of surrounding forests and wildlife, and livelihood context (National Statistics Office 2021). It has one of the highest human population densities (461/km^2^) in the world, with ongoing and planned large‐scale infrastructure development (Wikramanayake et al. 2010). Forests play a vital role in local livelihoods, with most households depending on forests for fuel, fodder and other resources (MOFSC 2015). Over 60% of households own less than a hectare of land, and the average annual per capita income is ~1500 USD (MOF 2025). Thus, remaining natural habitats are at risk of further fragmentation, increasing the proximity of tigers and humans leading to conflict (Gurung et al. 2008).

### 2.2 Human‐Tiger Conflict Data Collection

Data on human‐wildlife conflicts, including tiger attacks on human, are systematically registered at Protected Area Offices for incidents within park/reserves, buffer zones or conservation areas, whereas Division Forest Offices (DFO) for incidents outside the protected areas. Victims (or in fatal cases, their relatives) submit a written claim to access government relief claims (Dhakal et al. 2026). These records include details about the victims (name, age, sex, residential address), as well as specific information about the attacks, including the date and location of the incident site. For this study, we visited respective offices and compiled both the fatal and non‐fatal tiger attacks across the TAL from January 2010 to December 2024 and validated them with annual reports of respective offices.

To contextualize the distribution of tiger attacks relative to tiger occurrence, we used secondary data on tiger presence from the National Tiger Survey 2022. These data points represent confirmed tiger sightings or signs (pugmarks, scats, or camera trap detections) and provide a baseline for tiger distribution within the landscape during the study period.

### 2.3 Questionnaire Survey With the Victims Or Family Members

We conducted semi‐structured questionnaire surveys with the survivor of tiger attack or, in fatal cases, an adult family member familiar with the incident (*n* = 245) (File S1), to validate the data and gather additional contextual information not included in the official records about each incident. Respondents were identified from the PA/DFO relief‐claim records and contacted at household level via the local conservation officer or community forest user group representative familiar with the affected household.

Given the sensitive and potentially traumatic nature of the subject, we adopted an approach taking into account sensitivity to potential re‐traumatization. The questionnaire was designed to capture detailed insights into the circumstances of the attacks, including the location, time, activity of the victim, and group size at the time of attack, action taken and other relevant information specific to the incident (File S1). Before each interview, the purpose of the study, the voluntary nature of participation and the respondent's right to withdraw at any stage were explained and verbal consent was obtained. During survey: (i) all interviews were conducted in the respondent's preferred language (Nepali, Tharu or other local language as appropriate); (ii) respondents were given full control over pacing and were informed that they could pause or end the interview at any point; (iii) interviewers did not probe for incident detail beyond what respondents volunteered; and (iv) where respondents became discomforted or distressed, the interview was paused and resumed only with the respondent's consent. The survey was carried out by interviewing respondents by experienced and well‐trained interviewers (*n* = 5) all of whom were local citizen scientist with prior experience in conducting household surveys, participatory field research and shared the language and cultural context of respondents. Additionally, they were provided with a two‐day orientation by the first author. The first author was involved in household questionnaire interviews at the start to build confidence of the enumerators and verify consistency of survey administration before they continued independently. The questionnaire was pre‐tested with 20 respondents and revised before conducting the survey. The questionnaires were prepared in local language (Nepali) and interview was conducted with the consent of the respondent.

### 2.4 Data Analysis

All the data were entered in excel and personal information was removed to keep the identity of the interviewee and victims anonymous. We first categorized the temporal trend of tiger attacks on humans by years, seasons (months), and diurnal patterns (by hour). We also assessed the attack frequency in relation to victims' age and sex (Gurung et al. 2008; Kandel et al. 2023). We also summarized the data on group size of people during tiger attacks, their activity/posture, number of tiger attack incidents across protected areas and surrounding landscapes.

For our analysis in relation to fatality risk during a tiger attack we fitted a generalized linear model using a binomial likelihood distribution (died = 1, injured = 0) with a logit link (full model description in Files S2 and S3). Following explanatory variables were used: (1) Group (Victim with group of people (two or more) = 1, single = 0), (2) Victim's age (category 15–24, 25–44, 45–65, > 65) (Ram et al. 2021), (3) sex of the victim (male = 1, female = 0), (4) Ethnicity of victim 4‐category variable (Hill migrant, indigenous Terai, Dalit, and others), (5) Education of victim 2‐category variable (Secondary level or less, and higher secondary or more) (6) Activity of victim 6‐category variable (Agricultural work, Fodder/fuelwood collection, NTFP collection, livestock herding, HTC response and other), and (7) posture of victim during the attack as a 3‐category variable (upright = walking/standing, low lying = bending/sitting/sleeping, and unknown = no information about the posture of victim). To explore initial associations between individual categorical variables (sex, education, ethnicity, and activity) and the attack (fatality vs. injury), we performed Pearson's Chi‐square tests. In cases where expected cell frequencies were small (< 5), we employed Fisher's Exact tests with simulated *p*‐values (based on 2000 replicates) to ensure statistical rigor. Using multi‐model inference in the “MuMIn” package in R (Grueber et al. 2011), we ranked the best models based on AIC value (lower AIC value indicates higher model ranking). Final model parameter estimates, and predictions were obtained by averaging the top candidate models with delta AICc ≤ 2 (Burnham and Anderson 2004). Analysis was carried out in R (R Core Team 2025). All the analyses can be reproduced using the R‐script and the associated data provided in the Files (S2 and S3).

## 3 Results

### 3.1 Socioeconomic Characteristics of Tiger Attacked Victims

We documented a total of 245 tiger attacks (141 fatalities) in Nepal between 2010 and 2024. Males were attacked more frequently (65.3%, *n* = 160) compared to females while fatality rate was higher among females (72.9%, *n* = 62; (*χ* ^2^ = 11.67, df = 1, *p* < 0.001)). Majority of the victims (93%) had education of secondary level or below. Hill migrants were attacked more frequently (62%) compared to indigenous Terai communities. Tiger attacks occurred more frequently during routine forest‐based livelihood activities: fodder and firewood collection (54%), NTFP collection (11%) (Table 1).

**TABLE 1:** Socioeconomic characteristics, activity and postures of victims attacked by tigers in Nepal's Terai Arc Landscape between 2010 and 2024.

Category	Subcategory	Deaths	Injuries	Total	Fatality ratio (%)
Overall	All incidents	141	104	245	57.6
Sex	Male	79	81	160	49.4
	Female	62	23	85	72.9
Age group	15–24	6	4	10	60.0
	25–44	41	29	70	58.6
	45–64	41	34	75	54.7
	65+	14	7	21	66.7
Education	Secondary level or below	85	48	133	63.9
	High School or above	4	6	10	40.0
Ethnicity	Dalit	14	9	23	60.87
	Hill migrant	87	67	153	56.86
	Indigenous Terai	38	28	66	57.58
	Others	2	1	3	66.67
Activity of victim	Agricultural work	6	6	12	50.00
	Fishing	11	2	13	84.62
	Fodder and firewood collection	78	54	132	59.09
	Herding or livestock grazing	17	15	32	53.13
	Response during conflict incident	3	11	14	21.43
	NTFP collection	19	8	27	70.37
	Others	7	8	15	46.67
Posture of victim during attack	Bending or sitting	44	15	59	74.58
	Upright	29	42	71	40.85
	Unknown	68	47	115	59.13

### 3.2 Spatial and Temporal Patterns of Tiger Attacks

**FIGURE 2:**
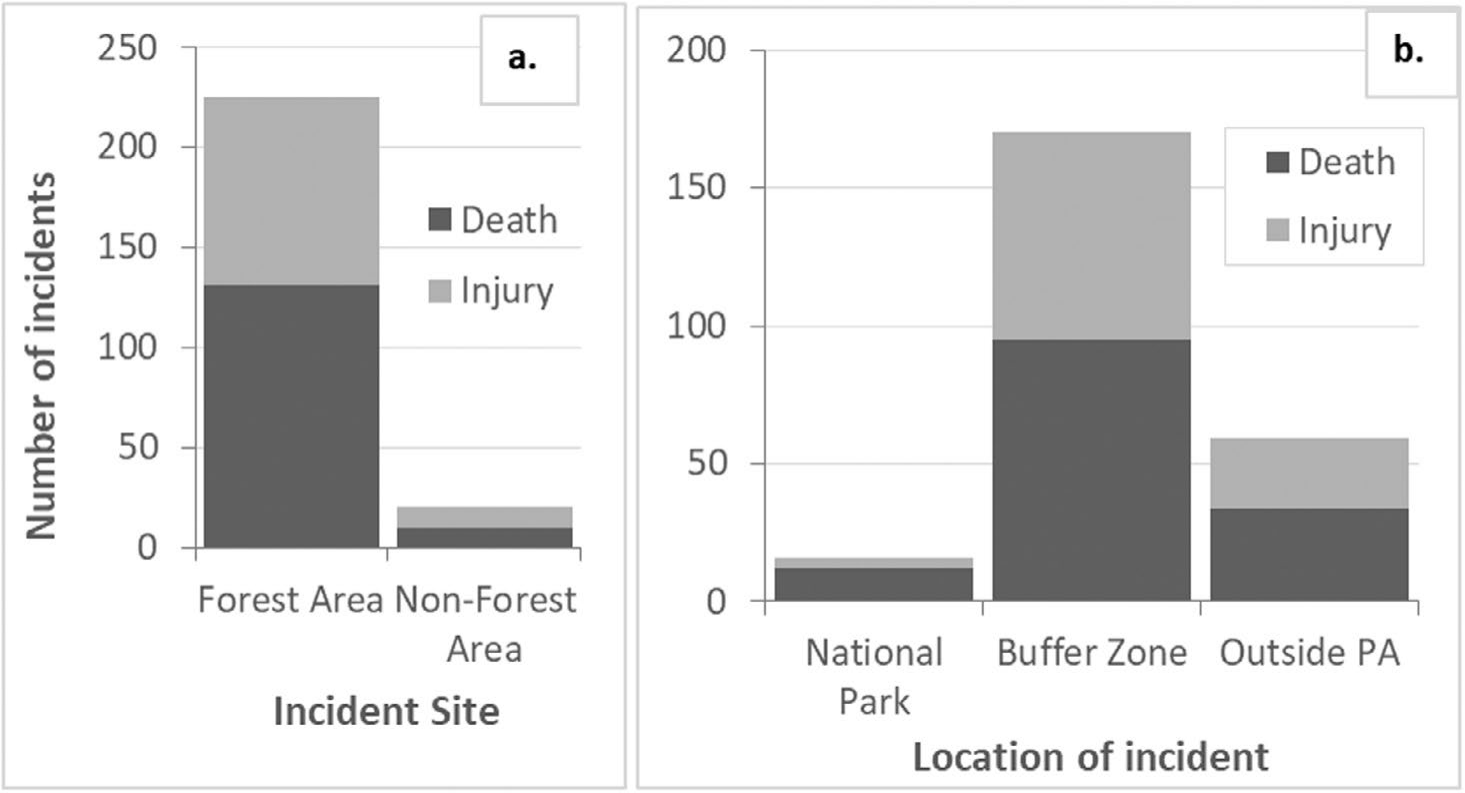
(a) Site category (forest, non‐forest) and (b) location (national park, buffer zone, or outside protected areas) of the tiger attacks on humans between 2010 and 2024 in TAL.

**FIGURE 3:**
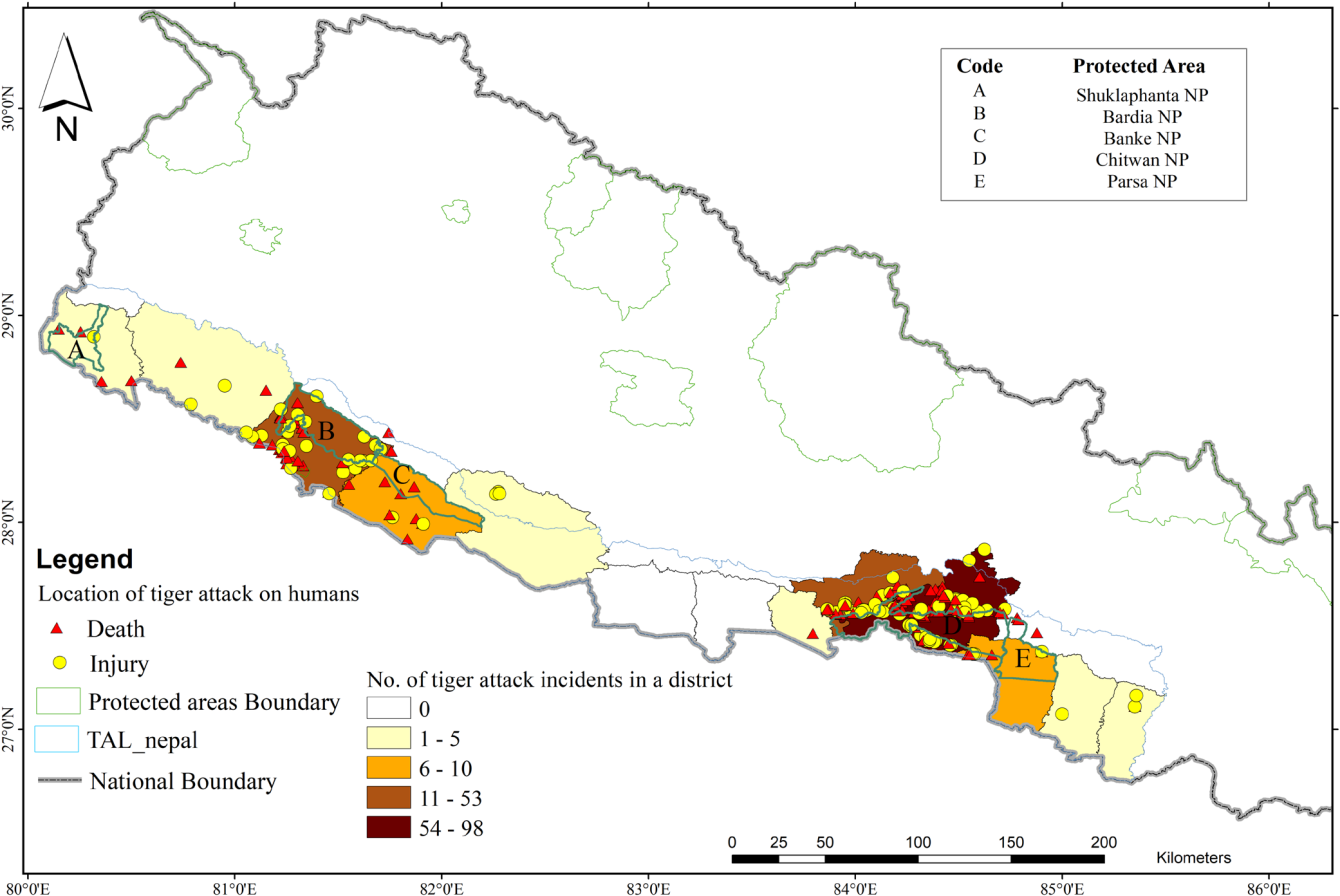
Locations of tiger attacks on human resulting in fatality (red triangles) and injury (yellow circles) in Nepal (2010–2024). Darker colors show higher intensity of attacks on a spatial scale of districts.

**FIGURE 4:**
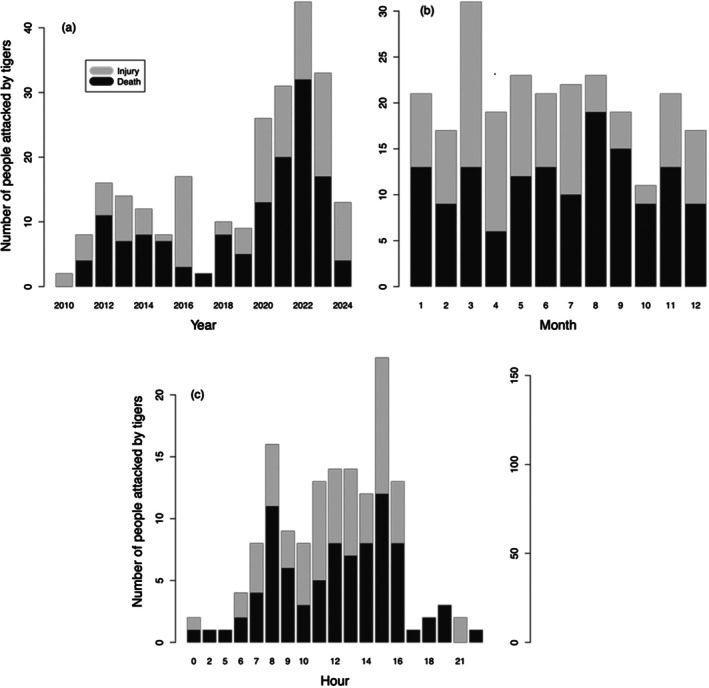
Temporal patterns of tiger attacks resulting in injury (gray) and death (black) at the scale of: (a) year, (b) month, and (c) hour during the 2010–2024 period in Nepal.

Over 90% of the tiger attacks happened inside forests (Table 1 and Figure 2). Over two thirds (69.4%) of the attacks occurred in buffer zones, followed by outside protected areas (24%) and only 16 incidents (6.5%) within the national parks (Figure 2). The highest number of tiger attacks (~47%, *n* = 245) occurred in the periphery of Chitwan National Park (Figure 3). However, tiger attacks increased sharply in the Banke‐Bardia complex and Far‐western regions during 2019–2023 (Figure 4a).

In Nepal, annual average tiger attacks on humans for the study period was 16 (±3.1 SE) with 57.6% resulting into fatalities. Attack frequency varied substantially among years, with the highest number of tiger attacks on human occurring during the COVID‐19 pandemic (Figure 4a). No distinct seasonal patterns in the tiger attacks were observed, although March showed a modest peak in total tiger attacks and August–September a slight peak in fatalities (Figure 4b). Most of the attacks happened during daytime (peak in the morning and afternoon) (Figure 4c).

### 3.3 Factors Influencing Risk of Fatality on Tiger Attack

Group size, sex, education and posture of victims during the tiger attack were associated with human fatality (Table 2). The risk of fatality was significantly lower when the victims were in groups (*p* < 0.001). Women were more vulnerable to fatality when attacked by tigers compared to men (*p* = 0.009). People with lower education (Secondary level or below) were more vulnerable to fatalities (*p* < 0.001). Similarly, activities in the upright position (standing or walking) had significantly lower risks of fatality compared to bending or sitting positions (*p* < 0.001).

**TABLE 2:** Variables associated with fatality risk on tiger attacks to humans in Nepal.

Parameters	Estimate	Std. error	*z*	Pr(>|*z*|)
(Intercept)	1.3296	0.6713	1.981	0.047645*
Group	−2.1235	0.3325	−6.386	1.70E‐10***
Sex_victimMale	−0.8995	0.3451	−2.607	0.009141**
Education_VictimHigh school or above	0.3748	0.9194	0.408	0.683527
Education_Victim Secondary level or below	2.0306	0.5863	3.463	0.000533***
PostureUnk	0.4923	0.6095	0.808	0.419274
Posture Upright	−1.7525	0.4573	−3.832	0.000127***

*** *p* < 0.001.

** *p* < 0.01.

* *p* < 0.05.

### 3.4 Community Perspectives on Causes and Mitigation of Tiger Attacks

Two third (67.2%) of the respondents believe that tigers come near settlements for “searching of prey” (i.e., tigers following wild prey or domestic livestock into settlement edges). The other reasons reported were conflict causing tiger habituated with people (12.3%), absence of physical barriers at the forest–settlement interface (5.7%), settlement proximity to dense forest (4.9%), incidental tiger movement (4.9%), increase in tiger population (2.5%), and habitat loss or degradation (2.5%) (File S4).

For reducing fatal human tiger encounters, majority of the respondents suggested adoption of safety measures, such as going into the forests in groups and remaining vigilant during forest product collection (56.2%), followed by avoidance of high‐risk areas or times (30.6%), protective infrastructure including fencing (11.1%) and ecological management (1.38%). Only a few respondents (0.71%) suggested lethal control of conflict‐causing tigers (File S4).

## 4 Discussion

This study presents landscape‐scale characterization of tiger attacks on humans in Nepal TAL, during the period of the tiger recovery between 2010 and 2024 (DNPWC and DFSC 2022). We documented the highest number of tiger attacks during the COVID‐19 pandemic, a pattern consistent with documented increases in human activities in the forest (Koju et al. 2021). Over 90% of tiger attacks on humans occurred within the forests and majority were within the buffer zones. Sex, posture, education and group size of the people were associated with the risk of fatalities when attacked by tigers (Dhanwatey et al. 2013; Paudel et al. 2024). Human fatality is therefore shaped by modifiable behavioral and social factors (Adler et al. 2026) such as posture during resource collection, and whether the person was alone.

### 4.1 Spatial and Temporal Pattern of Attacks

We documented the highest number of tiger attacks on humans during COVID‐19 coinciding with pandemic and its socioeconomic aftermath in the year 2022. Three concurrent processes plausibly contributed to increased chances for encounters with tigers (i) reverse migration of workers from Indian and Gulf labour markets, and the livelihood insecurity that followed (Maraseni et al. 2022) (ii) increased human activity in forested landscapes during lockdowns and the subsequent recovery period (Koju et al. 2021); and (iii) increase in illegal extraction of forest resources and wildlife poaching driven by lost wage income and unemployment (Koju et al. 2021; Maraseni et al. 2022). Most of the tiger attacks on humans in our study occurred inside the forest similar to Acharya et al. (2016). The number of attacks on humans has increased globally in the recent past due to habitat fragmentation/loss and/or increment of both human and large carnivore populations (Bombieri et al. 2023). Increasing outdoor activities in the developed world are attributed to increased human attacks by large carnivores (Penteriani et al. 2016). However, in Nepal, as in other developing countries, most of the attacks occurred during livelihood activities, primarily for resource collection/use in the forests (Gurung et al. 2008; Silwal et al. 2017; Bombieri et al. 2023). The attacks were high during the early morning and in the afternoon when livelihood activities in forests are at peak (Figure 4c), (Paudel et al. 2024). These patterns suggest that temporal access restrictions alone are not effective, but they need to align with seasonal and daily needs of forest‐dependent communities in high‐risk areas, accompanied by feasible alternatives (Dhanwatey et al. 2013; Ramesh et al. 2019).

Comparison of our findings with earlier studies indicates a clear geographic redistribution of human fatalities within TAL during the period of tiger recovery. Gurung et al. (2008) reported a tenfold increase in human killings by tigers and was mostly due to restoration of forests outside the national park in the buffer zone area of Chitwan (Mehta and Heinen 2001). We found a decrease in annual average fatalities compared to (Gurung et al. 2008) from 7.2 (±2.3 SE) persons during 1998–2006 to 4.53 (±1.1 SE) persons during 2010–2024 in Chitwan NP and buffer zone. However, the annual average human fatality for the study period in Nepal was higher (9.4 ± 2.15 SE) compared to the earlier period of 8.26 annual fatalities (1.06 and 7.2 persons/year in Bardia during 1994–2007 and Chitwan during 1998–2006 respectively) (Gurung et al. 2008; Bhattarai and Fischer 2014). Evidence from the Chitwan–Parsa Complex provides a context for this decline; Prasai et al. (2025) reported that 32 tigers were rescued from the buffer zones around Chitwan and Parsa between the start of 2020 and 2024, predominantly adult males.

Both the number of tiger attacks on humans and fatalities has substantially increased in Bardia NP and its surroundings after 2019 reaching a peak in 2022. As a response, various prevention and mitigation measures for tiger attacks on humans were implemented including capture and management of 21 conflict‐causing tigers during 2019–2024; behavior change campaign reaching a large number of people (Kadariya et al. 2023) and clearing undergrowth vegetation in buffer zones and corridors to reduce risks of tiger encounters. As a result, number of tiger attacks has decreased after 2023 in Bardia and surrounding areas (BNP 2025). Tiger numbers alone are an incomplete predictor of conflict, but social‐ecological conditions of exposure such as forest dependence, livelihood activity, and settlement history also affect tiger attacks on humans (Rastogi et al. 2014; Stevens et al. 2025).

Tiger attacks have been decreasing after 2022 which can be attributed to enhanced preventive and mitigative measures including management of conflict‐causing tigers (Lamichhane et al. 2017) and increasing awareness (Kadariya et al. 2023; Paudel et al. 2024). Therefore, a comprehensive system of regular monitoring, early detection and efficient removal of such conflict‐causing tigers should be established to avoid the chances of tiger attacks on humans (Gurung et al. 2008; Barlow et al. 2013; Lamichhane et al. 2017). Moreover, alternative livelihoods such as improved agriculture, stall feeding of livestock, skill‐based entrepreneurship, fish farms, tourist guides and homestays, which reduce the risk of human‐tiger confrontation should be promoted (Shahi et al. 2022).

Tiger density and HTC incidents appear to track one another over the decade within TAL, although the trend is not strictly linear and is mediated by the spatial distribution of tigers, prey abundance and human activity. The Chitwan tiger population was about 120 adults in 2013 while the tiger population in Bardia was 50 adults (Dhakal et al. 2014). The chances of encountering a tiger were minimal at that time in Bardia as density was relatively low. But while the tiger population reached about 100 adults (DNPWC and DFSC 2022) the number of tiger attacks on human surged in Bardia and periphery (Bhattarai and Fischer 2014; Paudel et al. 2024). It is similar to the escalation of fatalities in Chitwan after buffer zone restoration programs (Gurung et al. 2008). In recent years the tiger attacks outside protected areas have also increased with increasing tiger dispersal and colonization of these forests (DNPWC and DFSC 2022; Acharya et al. 2026).

We documented buffer zone as the hotspot for conflict, where over two thirds of the tiger attacks on humans occurred. The buffer zones in Nepal have defined boundaries around the national parks. The National Parks and Wildlife Conservation Act (1973) has provision of sharing 30%–50% of the protected area's revenue with the respective buffer zone communities for integrated conservation and community engagement programs (Lamichhane et al. 2019). The buffer zone forests, grasslands and rivers also support local livelihoods by providing access to natural resources. The buffer zone also provides additional contiguous habitat for wildlife. In recent years, tiger population has been increasing in the buffer zones (Acharya et al. 2026). Thus, special focus is required in the buffer zone for spatial and temporal separation between tiger and people to reduce their possible negative interactions (Treves and Karanth 2003; Goodrich 2010).

### 4.2 Human Fatality Risk

The risk of fatality was primarily affected by the sex, posture, education and group size of victims during the tiger attacks. Although men were attacked more frequently (65%), women were more vulnerable to fatalities (73%) compared to men (49%).

Women are often attacked during tasks that require vulnerable postures, such as bending or crouching to collect firewood, fodder, or minor forest products, which increases the lethality of encounters (Dhanwatey et al. 2013). These low body positions mimic prey to ambush predators like tigers, reducing detection and escape chances (Walter 2004). In contrast, men are typically attacked while standing or walking during cattle grazing, allowing better defensive responses. This division of labor, rooted in cultural norms and decision‐making translates into structurally unequal exposure to and consequences of human tiger conflict in areas like central India and Nepal (Doubleday and Adams 2020; Adler et al. 2026).

Silwal et al. (2017) documented higher proportion of women killed in Chitwan during fodder collection, which occurs in looser group formations with lower vigilance. Male‐biased attack frequency reflects men's guarding roles, yet women's higher fatality per attack highlights overlooked inequities (Treves and Naughton‐Treves 1999). During tiger attacks, risk of fatality decreased when people were in groups as vigilance increases. Immediate response by the colleagues and chasing tigers also reduces chances of fatality. Tigers are also scared of people when they are in groups (Shultz and Finlayson 2010).

The high frequency of Hill migrant victims of tiger attacks is in proportion to their share in the total population. For instance, population composition of three districts (Chitwan, Bardia and Nawalparasi East) with three‐fourths of the recorded tiger attacks comprised 61.7% hill‐migrants, 22.5% Indigenous Terai communities, 10.0% Dalit and 5.8% Other communities (NSO 2021). This indicates that all ethnic and social groups bear similar risks of tiger attacks in TAL.

### 4.3 Community Perspectives on Causes and Mitigation of Tiger Attacks

Community perception that tiger movement near settlements in search of prey is consistent with the concentration of tiger attacks on humans in buffer‐zone and forest‐edge areas where prey, people and tiger habitat overlap (Prasai et al. 2025). Tigers coming in fringe areas indicate the concentration of prey there, especially in recently restored buffer zone community forests and corridors (Wegge et al. 2018). The underlying reason for such pattern could be poor habitat in the core areas of national parks (Thapa et al. 2021). The communities also suggested creating physical barriers like fences at forest fringes to prevent tigers and other wildlife entering settlements and farmlands (Lamichhane et al. 2019). Real‐time tiger monitoring, early warning and rapid actions to identify and timely manage potentially conflict‐causing tigers are recommended by the communities to reduce conflict (Barlow et al. 2013; Dhungana et al. 2024).

The perspective of safer collective forest use and temporal or spatial avoidance strategies was suggested by nearly one third of the respondents. However, such spatial and temporal forest use avoidance or restrictions can transfer the disproportionate costs to forest‐dependent households, particularly women and economically marginalized communities, unless they are provided with accessible alternatives (Badola and Hussain 2003; Ogra 2008; Ramesh and Malviya 2015). Participatory planning can improve the contextual suitability and social legitimacy of mitigation measures by incorporating local knowledge, livelihood constraints and community priorities (Treves and Karanth 2003).

Socio‐ecological approaches that combine spatial estimates of encounter risk with the social determinants of tolerance can improve the prioritization and targeting of preventive interventions (Struebig et al. 2018). Sustained attention to the equitable distribution of conservation costs and benefits is therefore likely to be important for maintaining tolerance, securing participation in conflict‐reduction measures and reducing exposure to fatal encounters (Bhattarai et al. 2021).

## 5 Conservation Implications

Results of this study are useful for devising tiger conservation plans and human tiger conflict mitigation strategies in the human‐dominated landscape. As tigers continue to disperse outside national parks with restoration of degraded forest areas in buffer zone and beyond, the probability of negative human‐tiger interactions is likely to increase. Adaptive management of these forests focusing on spatial or temporal separation of humans and tigers should be prioritized to reduce risk of tiger attacks on humans through regular clearing of undergrowth vegetation to increase visibility, especially in high human‐use zones during monsoon and post‐monsoon season.

Adjusting human behavior adopting safety protocols while using the forests such as staying in groups, adjusting leaning postures, following notices and communications regarding the tigers' presence in the forests can reduce the risk of tiger attacks. Current interventions on mass awareness on behavior change and safety measures should be continued and scaled up. Regulating the entry of the forest users and visitors during early morning, late evening and restricting during night can minimize human tiger conflicts. Specific interventions that address the unique vulnerabilities of women should be prioritized to reduce their fatality risks.

Promoting alternative livelihood options for local communities such as homestays, tourist guides, agro‐based enterprises, skill‐based trainings could reduce forest dependency and chances of confrontation with tigers. Furthermore, promoting tiger tourism engaging local communities can foster human‐tiger coexistence. Timely response in case of tiger attacks and efficient relief payments for the victim's family are necessary for reducing community resentments. A comprehensive system of regular monitoring, early detection and efficient removal of conflict‐causing tigers will reduce the chances of tiger attacks on humans. In the buffer zone and beyond, community‐based tiger monitoring system can support timely identification of conflict‐causing tigers and their management.

## Author Contributions

**Bed Kumar Dhakal:** conceptualization (lead), data curation (lead), formal analysis (lead), funding acquisition (lead), investigation (lead), methodology (lead), writing – original draft (lead), writing – review and editing (lead). **Babu Ram Lamichhane:** formal analysis (equal), investigation (lead), methodology (equal), supervision (equal), writing – original draft (equal), writing – review and editing (equal). **Sanjay Kandel:** data curation (equal), formal analysis (equal), writing – original draft (equal). **Naresh Subedi:** conceptualization (equal), formal analysis (equal), investigation (equal), methodology (equal), supervision (lead), validation (lead), writing – original draft (equal), writing – review and editing (equal). **Matthew Low:** formal analysis (equal), writing – review and editing (equal). **Thakur Silwal:** conceptualization (equal), writing – original draft (equal).

## Funding

This study was supported by funding from the Zoological Society of London (ZSL) Nepal.

## Ethics Statement

This study involved semi‐structured questionnaire survey with victims of tiger attack and/or their adult family members. Ethical considerations were carefully addressed throughout the research process. Prior to each interview, the purpose of the study was clearly explained to the respondents, and verbal informed consent was obtained. Participation was entirely voluntary, and respondents were free to withdraw at any stage. No personal identifiers were disclosed, and all information was treated confidentially and used solely for research purposes. The study was conducted in accordance with ethical and legal requirements and was approved by the Department of National Parks and Wildlife Conservation, Nepal, with research permission letter No. 2851‐079/080 to access the relief complaint records.

## Conflicts of Interest

The authors declare no conflicts of interest.

## Supporting information


**File S1:** Questionnaire used for semi‐structured interview.

**File S2:** R Code for modeling fatality risk on tiger attack.

**File S3:** Victim characteristics and activity during tiger attacks. These variables were used to model the fatality risk (death_Injury) as function of various independent variables.

**File S4:** Synthesis of victims' and family members' perspectives on causes of tiger movement near settlements and measures for reducing tiger attacks, Nepal Terai Arc Landscape.

## Data Availability

Data are available in Zenodo Repository: https://doi.org/10.5281/zenodo.18766760.

## References

[ece374206-bib-0001] Acharya, H. B. , R. Amin , B. R. Lamichhane , et al. 2026. “Spatio‐Temporal Distribution of Tigers Across Different Management Zones and Its Conservation Implications In and Around Chitwan National Park, Nepal.” Oryx (In Press). http://www.oryxthejournal.org.

[ece374206-bib-0002] Acharya, K. P. , P. K. Paudel , S. R. Jnawali , P. R. Neupane , and M. Köhl . 2017. “Can Forest Fragmentation and Configuration Work as Indicators of Human–Wildlife Conflict? Evidences From Human Death and Injury by Wildlife Attacks in Nepal.” Ecological Indicators 80: 74–83. 10.1016/J.ECOLIND.2017.04.037.

[ece374206-bib-0003] Acharya, K. P. , P. K. Paudel , P. R. Neupane , and M. Köhl . 2016. “Human‐Wildlife Conflicts in Nepal: Patterns of Human Fatalities and Injuries Caused by Large Mammals.” PLoS One 11, no. 9: e0161717. 10.1371/journal.pone.0161717. 27612174 PMC5017643

[ece374206-bib-0004] Adler, K. A. , M. L. Gore , and C. E. Wilkinson . 2026. “The Gendered Costs of Human–Wildlife Conflict: A Global Systematic Review.” Ambio 55, no. 6: 1165–1180. 10.1007/s13280-025-02300-y. 41204041 PMC13125431

[ece374206-bib-0005] Aiyadurai, A. 2016. “Tigers Are Our Brothers: Understanding Human‐Nature Relations in the Mishmi Hills, Northeast India.” Conservation and Society 14, no. 4: 305–316. 10.4103/0972-4923.197614.

[ece374206-bib-0006] Badola, R. , and S. A. Hussain . 2003. “Conflict in Paradise: Women and Protected Areas in the Indian Himalayas.” Mountain Research and Development 23, no. 3: 234–237. 10.1659/0276-4741(2003)023[0234:CIP]2.0.CO;2.

[ece374206-bib-0007] Barlow, A. C. D. , I. Ahmad , and J. L. D. Smith . 2013. “Profiling Tigers ( *Panthera tigris* ) to Formulate Management Responses to Human‐Killing in the Bangladesh Sundarbans.” Wildlife Biology in Practice 9, no. 2: 30–39. 10.2461/wbp.2013.9.6.

[ece374206-bib-0008] Barua, M. , S. A. Bhagwat , and S. Jadhav . 2013. “The Hidden Dimensions of Human‐Wildlife Conflict: Health Impacts, Opportunity and Transaction Costs.” Biological Conservation 157: 309–316. 10.1016/j.biocon.2012.07.014.

[ece374206-bib-0009] Bhatt, T. R. , J. G. Castley , R. Sims‐Castley , H. S. Baral , and A. L. M. Chauvenet . 2023. “Connecting Tiger ( *Panthera tigris* ) Populations in Nepal: Identification of Corridors Among Tiger‐Bearing Protected Areas.” Ecology and Evolution 13, no. 5: e10140. 10.1002/ece3.10140. 37261321 PMC10227491

[ece374206-bib-0010] Bhattarai, B. R. , and K. Fischer . 2014. “Human‐Tiger *Panthera tigris* Conflict and Its Perception in Bardia National Park, Nepal.” Oryx 48, no. 4: 522–528. 10.1017/S0030605313000483.

[ece374206-bib-0011] Bhattarai, B. R. , D. Morgan , and W. Wright . 2021. “Equitable Sharing of Benefits From Tiger Conservation: Beneficiaries' Willingness to Pay to Offset the Costs of Tiger Conservation.” Journal of Environmental Management 284: 112018. 10.1016/J.JENVMAN.2021.112018. 33556825

[ece374206-bib-0079] BNP . 2025. Annual Report 2024/25. Bardia National Park Office.

[ece374206-bib-0012] Bombieri, G. , V. Penteriani , K. Almasieh , et al. 2023. “A Worldwide Perspective on Large Carnivore Attacks on Humans.” PLoS Biology 21, no. 1: e3001946. 10.1371/journal.pbio.3001946. 36719873 PMC9888692

[ece374206-bib-0013] Burnham, K. P. , and D. R. Anderson . 2004. Model Selection and Multimodel Inference. Springer New York. 10.1007/b97636.

[ece374206-bib-0014] Chapron, G. , P. Kaczensky , J. D. C. Linnell , et al. 2014. “Recovery of Large Carnivores in Europe's Modern Human‐Dominated Landscapes.” Science 346, no. 6216: 1517–1519. 10.1126/science.1257553. 25525247

[ece374206-bib-0015] Chapron, G. , D. G. Miquelle , A. Lambert , J. M. Goodrich , S. Legendre , and J. Clobert . 2008. “The Impact on Tigers of Poaching Versus Prey Depletion.” Journal of Applied Ecology 45, no. 6: 1667–1674. 10.1111/j.1365-2664.2008.01538.x.

[ece374206-bib-0016] Chowdhurym, A. N. , R. Mondal , A. Brahma , and M. K. Biswas . 2016. “Ecopsychosocial Aspects of Human–Tiger Conflict: An Ethnographic Study of Tiger Widows of Sundarban Delta, India.” Environmental Health Insights 10: 1–29. 10.4137/EHI.S24899. PMC4712980 26792997

[ece374206-bib-0017] Dhakal, B. K. , N. Subedi , B. R. Lamichhane , et al. 2026. “Human‐Wildlife Coexistence in Nepal: Evolution of Relief Policies and Practices.” Banko Janakari 36, no. 1: 83–98. 10.3126/banko.v36i1.78748.

[ece374206-bib-0018] Dhakal, M. , M. Karki , S. R. Jnawali , et al. 2014. “*Status of Tigers and Prey in Nepal, Department of National Parks and Wildlife Conservation; and Department of Forests and Soil Conservation, Government of Nepal*”.

[ece374206-bib-0019] Dhakal, N. P. , K. C. Nelson , and J. L. D. Smith . 2011. “Resident Well‐Being in Conservation Resettlement: The Case of Padampur in the Royal Chitwan National Park, Nepal.” Society & Natural Resources 24, no. 6: 597–615. 10.1080/08941921003709633.

[ece374206-bib-0020] Dhanwatey, H. S. , J. C. Crawford , L. A. S. Abade , P. H. Dhanwatey , C. K. Nielsen , and C. Sillero‐Zubiri . 2013. “Large Carnivore Attacks on Humans in Central India: A Case Study From the Tadoba‐Andhari Tiger Reserve.” Oryx 47, no. 2: 221–227. 10.1017/S0030605311001803.

[ece374206-bib-0021] Dhar, S. B. , and S. Mondal . 2023. “Nature of Human–Tiger Conflict in Indian Sundarban.” Trees, Forests and People 12: 100401. 10.1016/j.tfp.2023.100401.

[ece374206-bib-0022] Dhungana, R. , T. Maraseni , B. L. Allen , et al. 2024. “Multi‐Stakeholder Identification and Prioritization of Human‐Tiger Conflict Reduction Measures in Chitwan National Park, Nepal.” Oryx 58, no. 5: 655–663. 10.1017/S0030605323001734.

[ece374206-bib-0023] Dhungana, R. , T. Savini , J. B. Karki , M. Dhakal , B. R. Lamichhane , and S. Bumrungsri . 2018. “Living With Tigers *Panthera tigris* : Patterns, Correlates, and Contexts of Human‐Tiger Conflict in Chitwan National Park, Nepal.” Oryx 52, no. 1: 55–65. 10.1017/S0030605316001587.

[ece374206-bib-0024] DNPWC and DFSC . 2022. “*Status of Tigers and Prey in Nepal 2022, Department of National Parks and Wildlife Conservation, Kathmandu, Nepal*.” Kathmandu.

[ece374206-bib-0025] Doubleday, K. F. , and P. C. Adams . 2020. “Women's Risk and Well‐Being at the Intersection of Dowry, Patriarchy, and Conservation: The Gendering of Human–Wildlife Conflict.” Environment and Planning E: Nature and Space 3, no. 4: 976–998. 10.1177/2514848619875664.

[ece374206-bib-0026] Goodrich, J. M. 2010. “Human‐Tiger Conflict: A Review and Call for Comprehensive Plans.” Integrative Zoology 5, no. 4: 300–312. 10.1111/j.1749-4877.2010.00218.x. 21392348

[ece374206-bib-0027] Grueber, C. E. , S. Nakagawa , R. J. Laws , and I. G. Jamieson . 2011. “Multimodel Inference in Ecology and Evolution: Challenges and Solutions.” Journal of Evolutionary Biology 24: 699–711. 10.1111/j.1420-9101.2010.02210.x. 21272107

[ece374206-bib-0028] GTRP . 2011. “*Global Tiger Recovery Program 2010–2022*, *Global Tiger Initiative Secretariat*”.

[ece374206-bib-0029] Gurung, B. , J. L. D. Smith , C. McDougal , J. B. Karki , and A. Barlow . 2008. “Factors Associated With Human‐Killing Tigers in Chitwan National Park, Nepal.” Biological Conservation 141, no. 12: 3069–3078. 10.1016/j.biocon.2008.09.013.

[ece374206-bib-0030] Inskip, C. , and A. Zimmermann . 2009. “Human‐Felid Conflict: A Review of Patterns and Priorities Worldwide.” Oryx 43, no. 1: 18–34. 10.1017/S003060530899030X.

[ece374206-bib-0031] Jhala, Y. V. , N. A. Mungi , R. Gopal , and Q. Qureshi . 2025. “Tiger Recovery Amid People and Poverty.” Science 387: 505–510.

[ece374206-bib-0032] Kadariya, R. , K. C. R. Bahadur , U. Paudel , and B. P. Shrestha . 2023. “Behaviour Change Conservation Campaign for Improved Human–Tiger Coexistence in Nepal.” Oryx 57, no. 1: 11. 10.1017/S0030605322001363.

[ece374206-bib-0034] Kandel, S. , T. Silwal , S. K. Yadav , and S. Dhakal . 2023. “Temporal and Spatial Pattern of Wildlife Attacks on Human in Chitwan National Park, Nepal.” Nepalese Journal of Zoology 7, no. 1: 7–13. 10.3126/njz.v7i1.56305.

[ece374206-bib-0035] Karanth, K. U. , R. S. Chundawat , J. D. Nichols , and N. S. Kumar . 2004. “Estimation of Tiger Densities in the Tropical Dry Forests of Panna, Central India, Using Photographic Capture–Recapture Sampling.” Animal Conservation 7, no. 3: 285–290. 10.1017/S1367943004001477.

[ece374206-bib-0036] Koju, N. P. , R. C. Kandel , H. B. Acharya , B. K. Dhakal , and D. R. Bhuju . 2021. “COVID‐19 Lockdown Frees Wildlife to Roam but Increases Poaching Threats in Nepal.” Ecology and Evolution 11, no. 14: 9198–9205. 10.1002/ece3.7778. 34306616 PMC8293707

[ece374206-bib-0037] Lamichhane, B. R. , G. A. Persoon , H. Leirs , et al. 2017. “Are Conflict‐Causing Tigers Different? Another Perspective for Understanding Human‐Tiger Conflict in Chitwan National Park, Nepal.” Global Ecology and Conservation 11: 177–187. 10.1016/j.gecco.2017.06.003.

[ece374206-bib-0038] Lamichhane, B. R. , G. A. Persoon , H. Leirs , et al. 2018. “Spatio‐Temporal Patterns of Attacks on Human and Economic Losses From Wildlife in Chitwan National Park, Nepal.” PLoS One 13, no. 4: 1–18. 10.1371/journal.pone.0195373. PMC5908188 29672538

[ece374206-bib-0039] Lamichhane, B. R. , G. A. Persoon , H. Leirs , et al. 2019. “Contribution of Buffer Zone Programs to Reduce Human‐Wildlife Impacts: The Case of the Chitwan National Park, Nepal.” Human Ecology 47, no. 1: 95–110. 10.1007/s10745-019-0054-y.

[ece374206-bib-0040] Maraseni, T. , B. H. Poudyal , K. Aryal , and H. K. Laudari . 2022. “Impact of COVID‐19 in the Forestry Sector: A Case of Lowland Region of Nepal.” Land Use Policy 120: 106280. 10.1016/J.LANDUSEPOL.2022.106280. 35880191 PMC9300748

[ece374206-bib-0041] Mclean, J. , and S. Straede . 2003. “Conservation, Relocation, and the Paradigms of Park and People Management–A Case Study of Padampur Villages and the Royal Chitwan National Park, Nepal.” Society & Natural Resources 16, no. 6: 509–526. 10.1080/08941920309146.

[ece374206-bib-0042] Mehta, J. , and J. T. Heinen . 2001. “Does Community‐Based Conservation Shape Favorable Attitudes Among Locals? An Empirical Study From Nepal.” Environmental Management 28, no. 2: 165–177. 10.1007/s002670010215. 11443381

[ece374206-bib-0043] Miller, E. 2020. “More‐Than‐Human Agency: From the Human Economy to Ecological Livelihoods.” In The Handbook of Diverse Economies. Edward Elgar Publishing. 10.4337/9781788119962.00057.

[ece374206-bib-0044] MOF . 2025. “*Fiscal Survey 2081/82*”.

[ece374206-bib-0045] MOFSC . 2015. “*Strategy and Action Plan 2015–2025, Terai Arc Landscape, Nepal*.” Kathmandu.

[ece374206-bib-0046] National Statistics Office . 2021. National Population and Housing Census 2021. Government of Nepal. http://search.jamas.or.jp/link/ui/2019004882.

[ece374206-bib-0047] Nyhus, P. J. 2016. “Human‐Wildlife Conflict and Coexistence.” Annual Review of Environment and Resources 41: 143–171. 10.1146/annurev-environ-110615-085634.

[ece374206-bib-0048] Ogra, M. V. 2008. “Human–Wildlife Conflict and Gender in Protected Area Borderlands: A Case Study of Costs, Perceptions, and Vulnerabilities From Uttarakhand (Uttaranchal), India.” Geoforum 39, no. 3: 1408–1422. 10.1016/J.GEOFORUM.2007.12.004.

[ece374206-bib-0049] Pandey, H. P. , A. Apan , and T. N. Maraseni . 2026. “Assessing the Aftermath of Tripling the Tigers' Population in Nepal: Socio‐Economic and Eco‐Environmental Sustainability Perspectives.” Biodiversity and Conservation 35, no. 3: 96. 10.1007/s10531-026-03301-3.

[ece374206-bib-0050] Paudel, U. , R. B. Kc , R. Kadariya , et al. 2024. “Human–Wildlife Conflict in Bardia—Banke Complex: Patterns of Human Fatalities and Injuries Caused by Large Mammals.” Ecology and Evolution 14, no. 10. 10.1002/ece3.70395. PMC11461565 39385838

[ece374206-bib-0051] Penteriani, V. , M. M. Delgado , F. Pinchera , et al. 2016. “Human Behaviour Can Trigger Large Carnivore Attacks in Developed Countries.” Scientific Reports 6: 20552. 10.1038/srep20552. 26838467 PMC4738333

[ece374206-bib-0052] Prasai, A. , S. Lamichhane , A. Pathak , et al. 2025. “Tiger Habitat Occupancy in Chitwan–Parsa Complex: Implications for Human–Tiger Conflict Management Strategies.” Ecology and Evolution 15, no. 12: e72739. 10.1002/ece3.72739. 41426648 PMC12714492

[ece374206-bib-0053] R Core Team . 2025. “*R: A Language and Environment for Statistical Computing*.” Vienna, Austria: R Foundation for Statistical Computing. Accessed June 14, 2025. https://www.R‐project.org/.

[ece374206-bib-0054] Ram, A. K. , S. Mondol , N. Subedi , et al. 2021. “Patterns and Determinants of Elephant Attacks on Humans in Nepal.” Ecology and Evolution 11, no. 17: 11639–11650. 10.1002/ece3.7796. 34522330 PMC8427586

[ece374206-bib-0055] Ramesh, K. , and M. Malviya . 2015. “Human–Felid Conflict in Corridor Habitats: Implications for Tiger and Leopard Conservation in Terai Arc Landscape, India.” Human‐Wildlife Interactions, Spring 2015, Utah State University – Berryman Institute 9, no. 1: 48–57.

[ece374206-bib-0056] Ramesh, T. , R. Kalle , K. Sankar , Q. Qureshi , A. J. Giordano , and C. T. Downs . 2019. “To Resettle or Not?: Socioeconomic Characteristics, Livelihoods, and Perceptions Toward Resolving Human‐Tiger Conflict in the Nilgiri Biosphere Reserve, India.” Land Use Policy 83: 32–46. 10.1016/j.landusepol.2019.01.019.

[ece374206-bib-0057] Rastogi, A. , G. M. Hickey , R. Badola , and S. A. Hussain . 2014. “Understanding the Local Socio‐Political Processes Affecting Conservation Management Outcomes in Corbett Tiger Reserve, India.” Environmental Management 53, no. 5: 913–929. 10.1007/s00267-014-0248-4. 24522894

[ece374206-bib-0058] Rastogi, S. , P. Chanchani , M. Sankaran , and R. Warrier . 2022. “Grasslands Half‐Full: Investigating Drivers of Spatial Heterogeneity in Ungulate Occurrence in Indian Terai.” Journal of Zoology 316, no. 2: 139–153. 10.1111/jzo.12939.

[ece374206-bib-0059] Redpath, S. M. , J. Young , A. Evely , et al. 2013. “Understanding and Managing Conservation Conflicts.” Trends in Ecology & Evolution 28: 100–109. 10.1016/j.tree.2012.08.021. 23040462

[ece374206-bib-0060] Sanderson, E. W. , J. Moy , C. Rose , et al. 2019. “Implications of the Shared Socioeconomic Pathways for Tiger ( *Panthera tigris* ) Conservation.” Biological Conservation 231: 13–23. 10.1016/J.BIOCON.2018.12.017.

[ece374206-bib-0061] Seidensticker, J. , E. Dinerstein , S. P. Goyal , et al. 2010. “Tiger Range Collapse and Recovery at the Base of the Himalayas.” In Biology and Conservation of Wild Felids, edited by D. Macdonald and A. Loveridge , 305–324. Oxford University Press.

[ece374206-bib-0062] Shahi, K. , G. Khanal , R. R. Jha , A. K. Joshi , P. Bhusal , and T. Silwal . 2022. “Characterizing Damages Caused by Wildlife: Learning From Bardia National Park, Nepal.” Human Dimensions of Wildlife 27, no. 2: 173–182. 10.1080/10871209.2021.1890862.

[ece374206-bib-0063] Sharma, P. , N. Chettri , and K. Wangchuk . 2021. “Human–Wildlife Conflict in the Roof of the World: Understanding Multidimensional Perspectives Through a Systematic Review.” Ecology and Evolution 11, no. 17: 11569–11586. 10.1002/ece3.7980. 34522325 PMC8427619

[ece374206-bib-0064] Shultz, S. , and L. V. Finlayson . 2010. “Large Body and Small Brain and Group Sizes Are Associated With Predator Preferences for Mammalian Prey.” Behavioral Ecology 21, no. 5: 1073–1079. 10.1093/beheco/arq108.

[ece374206-bib-0065] Silwal, T. , J. Kolejka , B. P. Bhatta , S. Rayamajhi , R. P. Sharma , and B. S. Poudel . 2017. “When, Where and Whom: Assessing Wildlife Attacks on People in Chitwan National Park, Nepal.” Oryx 51, no. 2: 370–377. 10.1017/S0030605315001489.

[ece374206-bib-0066] Smith, J. L. D. , S. C. Ahearn , and C. McDougal . 1998. “Landscape Analysis of Tiger Distribution and Habitat Quality in Nepal.” Conservation Biology 12, no. 6: 1338–1346. 10.1111/j.1523-1739.1998.97068.x.

[ece374206-bib-0067] Stevens, M. , S. Rawat , and T. Satterfield . 2025. “Care, Conflict, and Coexistence: Human–Wildlife Relations in Community Forests.” People and Nature 7, no. 1: 231–246. 10.1002/pan3.10760.

[ece374206-bib-0068] Struebig, M. J. , M. Linkie , N. J. Deere , et al. 2018. “Addressing Human‐Tiger Conflict Using Socio‐Ecological Information on Tolerance and Risk.” Nature Communications 9, no. 1: 3455. 10.1038/s41467-018-05983-y. PMC6110717 30150649

[ece374206-bib-0069] Subedi, N. , S. R. Jnawali , M. Dhakal , et al. 2013. “Population Status, Structure and Distribution of the Greater One‐Horned Rhinoceros *Rhinoceros unicornis* in Nepal.” Oryx 47, no. 3: 352–360. 10.1017/S0030605313000562.

[ece374206-bib-0070] Subedi, N. , B. R. Lamichhane , Y. N. Dahal , M. Karki Thapa , R. Regmi , and B. Shrestha . 2021. “Tigers in the Himalayan Foothills: Possible Linkage Between Two Tiger Population Clusters in Terai Arc Landscape, Nepal.” Journal of Animal Diversity 3, no. 2: 69–75. 10.52547/JAD.2021.3.2.7.

[ece374206-bib-0071] Thapa, K. , and M. J. Kelly . 2017. “Density and Carrying Capacity in the Forgotten Tigerland: Tigers in the Understudied Nepalese Churia.” Integrative Zoology 12, no. 3: 211–227. 10.1111/1749-4877.12240. 27734629

[ece374206-bib-0072] Thapa, S. K. , J. F. Jong , N. Subedi , et al. 2021. “Forage Quality in Grazing Lawns and Tall Grasslands in the Subtropical Region of Nepal and Implications for Wild Herbivores.” Global Ecology and Conservation 30: e01747. 10.1016/j.gecco.2021.e01747.

[ece374206-bib-0073] Treves, A. , and K. U. Karanth . 2003. “Human‐Carnivore Conflict and Perspectives on Carnivore Management Worldwide.” Conservation Biology 17, no. 6: 1491–1499. 10.1111/j.1523-1739.2003.00059.x.

[ece374206-bib-0074] Treves, A. , and L. Naughton‐Treves . 1999. “Risk and Opportunity for Humans Coexisting With Large Carnivores.” Journal of Human Evolution 36, no. 3: 275–282. 10.1006/jhev.1998.0268. 10074384

[ece374206-bib-0075] Walter, M. 2004. “Defence of Bipedalism.” Human Evolution 19, no. 1: 19–44. 10.1007/BF02438907.

[ece374206-bib-0076] Wegge, P. , S. K. Yadav , and B. R. Lamichhane . 2018. “Are Corridors Good for Tigers *Panthera tigris* but Bad for People? An Assessment of the Khata Corridor in Lowland Nepal.” Oryx 52, no. 1: 35–45. 10.1017/S0030605316000661.

[ece374206-bib-0077] Wikramanayake, E. , A. Manandhar , S. Bajimaya , S. Nepal , G. Thapa , and K. Thapa . 2010. “The Terai Arc Landscape.” In Tigers of the World, 163–173. Elsevier. 10.1016/B978-0-8155-1570-8.00010-4.

[ece374206-bib-0078] Wikramanayake, E. , E. Wikramanayake , M. McKNIGHT , et al. 2004. “Designing a Conservation Landscape for Tigers in Human‐Dominated Environments.” Conservation Biology 18, no. 3: 839–844. 10.1111/J.1523-1739.2004.00145.X.

