# Supplementary material for: Tigers in the Neighborhood: Factors Associated With Fatal Tiger Attacks in a Human‐Dominated Landscape, Nepal
Source: Ecol Evol. 2026 Aug 16;16(8):e74206. doi: 10.1002/ece3.74206 (PMC13478372; doi:10.1002/ece3.74206)
Supplement: Supplementary file 1 [file ECE3-16-e74206-s001.docx]

**Tigers in the neighborhood: Factors associated with fatal tiger attacks in a human-dominated landscape, Nepal**

Bed Kumar Dhakal, Babu Ram Lamichhane, Sanjay Kandel, Naresh Subedi, Matthew Low, Thakur Silwal

Supplementary File S1 – Questionnaire used for semi-structured interview.

**Interviewer:	Date of interview (A. D.):**

1. **General information of respondent**

Name:	Age:	Sex: (M/F)

**Address:** District:	Municipality:	Ward No:	Tole:

GPS location of interview (House location):

Education: (Master and above, Bachelor, SLC, Simple literate, Illiterate)

Your relation to the victim: **Self, Family member, relative, neighbor**

2. **Detail of victims on tiger attack**

Casualty type:	a) Death	b) Severe injury	c) Minor injury

Name of victim:

Sex (Male/Female)	Education: Illiterate / Literate / SLC / College or above

Occupation:

Cast/Ethnicity:	1) Hill migrant 2) Ethnic 3) Indigenous Terai 4) Dalit and 5) Others

Marital/family status: a) Unmarried	b) Married – no children	c) Married & have children

Victim family member number:

Relation of respondent with victim:

Major income source of victim's family:

Land holding size (…………..Bigha/Katha)

3. **Incident details**

- Date (In A.D.):

Year	Month	Day	time: am/pm

- Incident area/ GPS location :
- Victim's activity during attack:
  a. Stay at house or periphery
  b. Working in agriculture field
  c. Cattle grazing in forests
  d. Fodder collection
  e. Collection of fern
  f. Collection of asparagus
  g. Collection of mushroom
  h. Collection of firewood
  i. Collection of timber
  j. Fishing
  k. Walking on the way
  l. If other please specify
- Location of attacked site : Grass land/Sparse River rain/Shrub and bush/Other land (please specify)
- What was the posture of victim during the tiger attack?

(Lie down/Sleeping/Standing/Walking/Sitting/Others please specify)

- From which side tiger attacked the victim? (From front/From side/From back)
- Please describe further if you know more about the tiger attack.

4. Do you have community forest? Yes/No
5. If yes what are the provision of collection of forest products? (Which product in which date?)
6. What is the provision of enter into community forest? (Alone or group)?
7. Was victim alone or in a group? If in group how many person were there? Did they cry or not?
8. Do they have any domestic arm (Stick/Knife/or other) and if he/she fought with tiger or not?
9. Which color cloth did victim wear during attack?
10. How the incident was reported?
11. How did victim rescue? Who did the rescue work?
12. What is the role of CBAPU during rescue operation?
13. Have the victim been taken to hospital? (Yes/No)
14. If yes by which transportation means? (Ambulance/Motorcycle/Taxi/Public bus)
15. What is the condition of injured person right now? (Totally recovered/partially disabled/Disable)
16. How much money did the victim expense for treatment?
17. In case of death of the victim, where was location of death?

(On the tiger attack spot, on the way to hospital, after discharge of hospital (within 30 days) and after one month of hospital discharge

18. Did the victim or family member receive some financial support from community forest?
19. Did the family receive relief from Government?
20. If yes please mention the relief support amount? How much time does take place to provide relief in months?
21. For what activities did victim family use the received relief money?
    a. Children education
    b. Land purchase
    c. Construction of new house
    d. Payment of loan
    e. Deposit in the bank
    f. Others (please specify)
22. Have the family received any additional relief or livelihood/education support? Yes	No

If yes give details with amount and quantity (From Local government, NGO, and other organizations)

23. What is the household social consequences from this incident?
    a. Who are the bread earners in the family?
    b. Was the victim have small children (Below 16 years)?
    c. Impact on children's education and welfare
    d. Economic impact in the house
    e. Psychological trauma and its consequences
    f. Social impacts (widowed, re-marriage, its effect on children)
    g. Other effects
24. Do the injured person involve in conservation? (If he/she involves in any community based organization please specify).
25. What are the measures to minimize the tiger attack in community forest?
    a. Zoning in community forest and strictly follow of treatment according to zones
    b. Do not manage habitat for wildlife but manage for optimum production of forest products
    c. Both habitat management and forest product use activities go together
    d. Strict follow up of forest entry date and time
    e. Others (please specify)

**Additional information**

26. Do you have livestock?	Yes	No
27. If yes how do you ranch livestock?
    a. Stall feeding	b. Grazing
28. From where do you collect grass for stall feeding?
29. Where is the grazing sites?
30. How frequently do you enter into forests other than community forests? A) Do not enter	b) Occasionally (less than 4 times a month), c) Frequently (once a week), d) regularly (more than four days in a week)
31. If you go forests frequently/regularly please mention your time?

morning/day/evening/night

32. While going to park are you going alone or you go with other friends? (Alone/in group)
33. Do you have any encounter with a tiger on such occasion? (Yes/No) If yes at what situation did you see the tiger? What you did immediately after seeing the tiger?
34. Is there any case of appearing the problem tiger in your village? (Yes, No) If yes please give detail of when it was appear? (Date, male/female, age, location and other information)
35. Why did tiger come into settlement area?
36. What are the measures to prevent tiger entering into settlement?
37. How did the problem tiger control? (Captured from park authorities in coordination with NTNC, captured from DFO authorities in coordination with PA and NTNC, killed by local, poisoning of tiger, others please specify)?
38. What should be done for the people attacking tigers?
    a. Kill
    b. Capture and put in enclosure
    c. Leave it in the jungle
    d. Others measure (Keep in Zoos of Nepal, Gift to zoo of other country for conservation education, Gift to other countries to release in nature)
39. Do you still want tigers to occur in your forest?	Yes	No

Why?

40. Where do you think tigers should occur?
    a. Tigers should be conserved in all forests
    b. Tigers should be conserved in the park only
    c. Tigers should be put in zoo only
    d. All tigers should be removed from forests including from parks
41. What do you suggest to avoid/minimize the human tiger conflict?
42. What do you suggest to make relief program more effective and efficient?
43. What do you think the role of local government, province government and central government to minimize the human tiger conflict and also provide relief amount? Please give in detail.
44. Do you have any ideas of involvement of NGOs, CBOs and other organizations in minimizing human tiger conflict?
45. Do you have anything to say about minimizing human tiger conflict? Please elaborate.

Thank you for your cooperation and support.
