# Supplementary material for: Tigers in the Neighborhood: Factors Associated With Fatal Tiger Attacks in a Human‐Dominated Landscape, Nepal
Source: Ecol Evol. 2026 Aug 16;16(8):e74206. doi: 10.1002/ece3.74206 (PMC13478372; doi:10.1002/ece3.74206)
Supplement: Supplementary file 2 [file ECE3-16-e74206-s002.docx]

**Tigers in the neighborhood: Factors associated with fatal tiger attacks in a human-dominated landscape, Nepal**

Bed Kumar Dhakal, Babu Ram Lamichhane, Sanjay Kandel, Naresh Subedi, Matthew Low, Thakur Silwal

Supplementary file S3. Victim characteristics and activity during tiger attacks. These variables were used to model the fatality risk (death_Injury) as function of various independent variables.

| Respondent ID | Death_injury | Sex_victim | Age_Victim | Age_category | Education_Victim | Ethnicity_victim | Activity | Group | Posture |
| --- | --- | --- | --- | --- | --- | --- | --- | --- | --- |
| ETC_1 | 0 | Male | 68 | 65+ | Secondary level or below | Hill Ethnic | HTC response | 1 | Upright |
| ETC_2 | 1 | Male | 43 | 25-44 | Secondary level or below | Hill Migrant | Fodder-fuelwood | 0 | Low lying |
| ETC_3 | 0 | Male | 46 | 45-64 | Secondary level or below | Hill Ethnic | Fodder-fuelwood | 1 | Upright |
| ETC_4 | 0 | Male | 58 | 45-64 | Secondary level or below | Indigenous Terai | HTC response | 0 | Upright |
| ETC_5 | 0 | Female | 50 | 45-64 | Secondary level or below | Hill Ethnic | Fodder-fuelwood | 1 | Upright |
| ETC_6 | 1 | Female | 56 | 45-64 | Secondary level or below | Hill Ethnic | Fodder-fuelwood | 0 | Low lying |
| ETC_7 | 0 | Male | 62 | 45-64 | Secondary level or below | Indigenous Terai | Livestock herding | 0 | Upright |
| ETC_8 | 0 | Male | 20 | 15-24 | High school or above | Hill Migrant | Agricultural Work | 0 | Low lying |
| ETC_9 | 1 | Male | 17 | 15-24 | Secondary level or below | Hill Ethnic | HTC response | 0 | Unk |
| ETC_10 | 1 | Female | 53 | 45-64 | Secondary level or below | Hill Ethnic | Fodder-fuelwood | 0 | Low lying |
| ETC_11 | 1 | Female | 39 | 25-44 | Secondary level or below | Hill Ethnic | NTFP colletion | 1 | Low lying |
| ETC_12 | 1 | Male | 63 | 45-64 | Secondary level or below | Dalit | Others | 0 | Upright |
| ETC_13 | 0 | Male | 40 | 45-64 | Secondary level or below | Hill Ethnic | Livestock herding | 1 | Upright |
| ETC_14 | 1 | Male | 25 | 25-44 | Secondary level or below | Indigenous Terai | NTFP colletion | 1 | Upright |
| ETC_15 | 1 | Female | 24 | 15-24 | Secondary level or below | Indigenous Terai | NTFP colletion | 0 | Low lying |
| ETC_16 | 1 | Male | 35 | 25-44 | High school or above | Hill Ethnic | Fodder-fuelwood | 0 | Unk |
| ETC_17 | 1 | Male | 61 | 45-64 | Secondary level or below | Hill Migrant | Fodder-fuelwood | 0 | Low lying |
| ETC_18 | 0 | Male | 40 | 25-44 | Secondary level or below | Hill Ethnic | Fodder-fuelwood | 1 | Low lying |
| ETC_19 | 1 | Male | 55 | 45-64 | Secondary level or below | Hill Migrant | Fodder-fuelwood | 0 | Low lying |
| ETC_20 | 1 | Male | 65 | 65+ | Secondary level or below | Hill Migrant | Fodder-fuelwood | 1 | Low lying |
| ETC_21 | 0 | Female | 45 | 45-64 | Secondary level or below | Hill Ethnic | Fodder-fuelwood | 1 | Low lying |
| ETC_22 | 1 | Male | 66 | 65+ | Secondary level or below | Hill Migrant | Fodder-fuelwood | 0 | Unk |
| ETC_23 | 1 | Male | 63 | 45-64 | Secondary level or below | Hill Migrant | Fodder-fuelwood | 0 | Low lying |
| ETC_24 | 1 | Female | 20 | 15-24 | Secondary level or below | Hill Migrant | NTFP colletion | 1 | Low lying |
| ETC_25 | 1 | Female | 60 | 45-64 | Secondary level or below | Dalit | Fodder-fuelwood | 0 | Unk |
| ETC_26 | 1 | Male | 40 | 25-44 | High school or above | Indigenous Terai | Fishing | 0 | Low lying |
| ETC_27 | 1 | Male | 40 | 25-44 | Secondary level or below | Hill Ethnic | Livestock herding | 0 | Unk |
| ETC_28 | 0 | Female | 25 | 25-44 | Secondary level or below | Hill Ethnic | NTFP colletion | 0 | Upright |
| ETC_29 | 0 | Female | 39 | 25-44 | Secondary level or below | Hill Ethnic | NTFP colletion | 1 | Low lying |
| ETC_30 | 1 | Male | 27 | 25-44 | Secondary level or below | Hill Ethnic | Fodder-fuelwood | 0 | Upright |
| ETC_31 | 1 | Male | 47 | 45-64 | Secondary level or below | Hill Ethnic | Fodder-fuelwood | 1 | Upright |
| ETC_32 | 0 | Male | 23 | 15-24 | Secondary level or below | Indigenous Terai | HTC response | 1 | Upright |
| ETC_33 | 0 | Male | 40 | 25-44 | Secondary level or below | Hill Migrant | Livestock herding | 1 | Upright |
| ETC_34 | 1 | Male | Unk | Unk | Secondary level or below | Dalit | Agricultural Work | 0 | Low lying |
| ETC_35 | 1 | Male | Unk | Unk | Secondary level or below | Hill Ethnic | HTC response | 1 | Low lying |
| ETC_36 | 0 | Male | 51 | 45-64 | Secondary level or below | Hill Migrant | Others | 0 | Upright |
| ETC_37 | 1 | Male | Unk | Unk | Secondary level or below | Hill Migrant | Fodder-fuelwood | 1 | Upright |
| ETC_38 | 1 | Male | Unk | Unk | Secondary level or below | Hill Ethnic | NTFP colletion | 0 | Unk |
| ETC_39 | 0 | Female | 58 | 45-64 | Secondary level or below | Dalit | Fodder-fuelwood | 0 | Low lying |
| ETC_40 | 1 | Female | 25 | 25-44 | Secondary level or below | Hill Migrant | Fodder-fuelwood | 1 | Low lying |
| ETC_41 | 1 | Male | 43 | 25-44 | Secondary level or below | Dalit | NTFP colletion | 0 | Low lying |
| ETC_42 | 1 | Female | 43 | 25-44 | Secondary level or below | Indigenous Terai | NTFP colletion | 1 | Low lying |
| ETC_43 | 1 | Female | 34 | 25-44 | Secondary level or below | Hill Migrant | Fodder-fuelwood | 1 | Unk |
| ETC_44 | 0 | Male | 70 | 65+ | Secondary level or below | Hill Migrant | Fodder-fuelwood | 1 | Low lying |
| ETC_45 | 0 | Male | 73 | 65+ | High school or above | Hill Migrant | NTFP colletion | 0 | Upright |
| ETC_46 | 0 | Male | 52 | 45-64 | Secondary level or below | Indigenous Terai | NTFP colletion | 1 | Upright |
| ETC_47 | 0 | Female | 29 | 25-44 | Secondary level or below | Hill Migrant | Agricultural Work | 0 | Upright |
| ETC_48 | 0 | Male | 36 | 25-44 | High school or above | Indigenous Terai | Fodder-fuelwood | 1 | Low lying |
| ETC_49 | 1 | Female | Unk | Unk | Secondary level or below | Indigenous Terai | NTFP colletion | 1 | Low lying |
| ETC_50 | 1 | Male | Unk | Unk | Secondary level or below | Indigenous Terai | Fishing | 1 | Low lying |
| ETC_51 | 1 | Female | Unk | Unk | Secondary level or below | Indigenous Terai | Fishing | 1 | Low lying |
| ETC_52 | 1 | Male | Unk | Unk | Secondary level or below | Indigenous Terai | Fishing | 1 | Low lying |
| ETC_53 | 1 | Female | Unk | Unk | Secondary level or below | Indigenous Terai | Fishing | 1 | Upright |
| ETC_54 | 1 | Male | Unk | Unk | Secondary level or below | Indigenous Terai | Fodder-fuelwood | 1 | Low lying |
| ETC_55 | 1 | Female | Unk | Unk | Secondary level or below | Indigenous Terai | Fodder-fuelwood | 1 | Upright |
| ETC_56 | 1 | Female | Unk | Unk | Secondary level or below | Indigenous Terai | NTFP colletion | 1 | Low lying |
| ETC_57 | 0 | Male | 71 | 65+ | Secondary level or below | Hill Migrant | Fodder-fuelwood | 0 | Upright |
| ETC_58 | 0 | Male | 53 | 45-64 | Secondary level or below | Indigenous Terai | HTC response | 1 | Upright |
| ETC_59 | 0 | Male | Unk | Unk | Secondary level or below | Indigenous Terai | HTC response | 1 | Upright |
| ETC_60 | 0 | Male | Unk | Unk | Secondary level or below | Indigenous Terai | HTC response | 1 | Upright |
| ETC_61 | 0 | Female | Unk | Unk | Secondary level or below | Hill Migrant | Fodder-fuelwood | 1 | Upright |
| ETC_62 | 0 | Male | 46 | 45-64 | Secondary level or below | Hill Migrant | HTC response | 1 | Upright |
| ETC_63 | 0 | Male | 46 | 45-64 | Secondary level or below | Hill Ethnic | Fodder-fuelwood | 1 | Upright |
| ETC_64 | 0 | Female | 53 | 45-64 | Secondary level or below | Hill Migrant | HTC response | 1 | Upright |
| ETC_65 | 1 | Male | Unk | Unk | Secondary level or below | Hill Migrant | NTFP colletion | 1 | Low lying |
| ETC_66 | 1 | Female | Unk | Unk | Secondary level or below | Hill Ethnic | Fodder-fuelwood | 1 | Low lying |
| ETC_67 | 0 | Male | 44 | 25-44 | Secondary level or below | Indigenous Terai | HTC response | 0 | Upright |
| ETC_68 | 1 | Female | 50 | 45-64 | Secondary level or below | Hill Ethnic | Livestock herding | 0 | Upright |
| ETC_69 | 0 | Female | 50 | 45-64 | Secondary level or below | Hill Migrant | Fodder-fuelwood | 0 | Low lying |
| ETC_70 | 0 | Male | 58 | 45-64 | Education_Unk | Dalit | Agricultural Work | 0 | Upright |
| ETC_71 | 0 | Male | 49 | 45-64 | Secondary level or below | Hill Ethnic | Livestock herding | 0 | Upright |
| ETC_72 | 1 | Male | 35 | 25-44 | Secondary level or below | Dalit | NTFP colletion | 1 | Low lying |
| ETC_73 | 1 | Female | Unk | Unk | Secondary level or below | Hill Ethnic | Fodder-fuelwood | 0 | Low lying |
| ETC_74 | 0 | Male | 39 | 25-44 | Secondary level or below | Indigenous Terai | Agricultural Work | 0 | Upright |
| ETC_75 | 0 | Male | 41 | 25-44 | Secondary level or below | Indigenous Terai | HTC response | 1 | Upright |
| ETC_76 | 0 | Female | 53 | 45-64 | Secondary level or below | Hill Migrant | Fodder-fuelwood | 0 | Upright |
| ETC_77 | 0 | Male | 54 | 45-64 | Secondary level or below | Indigenous Terai | Livestock herding | 0 | Upright |
| ETC_78 | 0 | Male | 38 | 25-44 | Secondary level or below | Hill Migrant | Others | 1 | Upright |
| ETC_79 | 1 | Male | 64 | 45-64 | Secondary level or below | Hill Migrant | Livestock herding | 0 | Unk |
| ETC_80 | 0 | Female | Unk | Unk | Secondary level or below | Hill Migrant | Livestock herding | 1 | Upright |
| ETC_81 | 0 | Male | 51 | 45-64 | Secondary level or below | Hill Ethnic | Others | 1 | Upright |
| ETC_82 | 0 | Male | 56 | 45-64 | Secondary level or below | Hill Migrant | Livestock herding | 1 | Upright |
| ETC_83 | 0 | Male | 35 | 25-44 | Secondary level or below | Hill Migrant | Livestock herding | 1 | Upright |
| ETC_84 | 0 | Male | 49 | 45-64 | Secondary level or below | Hill Ethnic | Livestock herding | 1 | Upright |
| ETC_85 | 0 | Male | 36 | 25-44 | Secondary level or below | Indigenous Terai | Fodder-fuelwood | 1 | Low lying |
| ETC_86 | 1 | Male | Unk | Unk | Secondary level or below | Indigenous Terai | Others | 0 | Unk |
| ETC_87 | 0 | Male | 72 | 65+ | Secondary level or below | Hill Migrant | NTFP colletion | 1 | Low lying |
| ETC_88 | 1 | Male | 69 | 65+ | Secondary level or below | Hill Migrant | Livestock herding | 0 | Upright |
| ETC_89 | 1 | Male | Unk | Unk | Secondary level or below | Hill Ethnic | Fodder-fuelwood | 0 | Unk |
| ETC_90 | 1 | Male | Unk | Unk | Secondary level or below | Dalit | Fishing | 0 | Low lying |
| ETC_91 | 1 | Male | Unk | Unk | Secondary level or below | Indigenous Terai | Fodder-fuelwood | 1 | Upright |
| ETC_92 | 1 | Male | Unk | Unk | Secondary level or below | Hill Ethnic | Others | 1 | Upright |
| ETC_93 | 1 | Male | Unk | Unk | Secondary level or below | Hill Ethnic | Fodder-fuelwood | 1 | Upright |
| ETC_94 | 1 | Male | Unk | Unk | Secondary level or below | Hill Ethnic | Fodder-fuelwood | 1 | Unk |
| ETC_95 | 1 | Female | Unk | Unk | High school or above | Hill Migrant | Fodder-fuelwood | 1 | Unk |
| ETC_96 | 0 | Male | 60 | 45-64 | Secondary level or below | Hill Ethnic | Fodder-fuelwood | 1 | Low lying |
| ETC_97 | 1 | Male | Unk | Unk | Secondary level or below | Hill Ethnic | Fodder-fuelwood | 1 | Low lying |
| ETC_98 | 0 | Male | 59 | 45-64 | Secondary level or below | Hill Migrant | Fodder-fuelwood | 1 | Upright |
| ETC_99 | 1 | Female | 59 | 45-64 | Secondary level or below | Indigenous Terai | NTFP colletion | 0 | Unk |
| ETC_100 | 1 | Male | 60 | 45-64 | Secondary level or below | Hill Migrant | Fodder-fuelwood | 1 | Unk |
| ETC_101 | 1 | Male | 68 | 65+ | Education_Unk | Hill Migrant | Others | 0 | Unk |
| ETC_102 | 1 | Female | 22 | 15-24 | Secondary level or below | Hill Migrant | NTFP colletion | 1 | Unk |
| ETC_103 | 1 | Male | Unk | Unk | Secondary level or below | Hill Ethnic | Agricultural Work | 0 | Low lying |
| ETC_104 | 1 | Female | Unk | Unk | Secondary level or below | Hill Migrant | Fodder-fuelwood | 0 | Low lying |
| ETC_105 | 1 | Female | Unk | Unk | Secondary level or below | Hill Ethnic | Fodder-fuelwood | 0 | Unk |
| ETC_106 | 1 | Female | 72 | 65+ | Secondary level or below | Hill Ethnic | Fodder-fuelwood | 0 | Unk |
| ETC_107 | 1 | Male | 32 | 25-44 | Education_Unk | Hill Migrant | Fodder-fuelwood | 0 | Unk |
| ETC_108 | 1 | Male | 63 | 25-64 | Education_Unk | Hill Ethnic | Fodder-fuelwood | 0 | Unk |
| ETC_109 | 0 | Male | Unk | Unk | Education_Unk | Hill Migrant | Fodder-fuelwood | 1 | Unk |
| ETC_110 | 1 | Female | 59 | 45-64 | Education_Unk | Dalit | Fodder-fuelwood | 0 | Unk |
| ETC_111 | 1 | Female | 36 | 25-44 | Education_Unk | Hill Ethnic | Fodder-fuelwood | 0 | Unk |
| ETC_112 | 1 | Male | 37 | 25-44 | Education_Unk | Hill Migrant | Others | 0 | Unk |
| ETC_113 | 1 | Female | 25 | 15-24 | Education_Unk | Hill Migrant | Fodder-fuelwood | 0 | Unk |
| ETC_114 | 0 | Male | 26 | 25-44 | Education_Unk | Dalit | HTC response | 0 | Unk |
| ETC_115 | 1 | Male | 64 | 45-64 | Education_Unk | Hill Migrant | Livestock herding | 0 | Unk |
| ETC_116 | 0 | Male | 55 | 45-64 | Education_Unk | Hill Ethnic | Fodder-fuelwood | 1 | Unk |
| ETC_117 | 1 | Female | 63 | 45-64 | Education_Unk | Hill Migrant | Fodder-fuelwood | 0 | Unk |
| ETC_118 | 1 | Male | 66 | 65+ | Education_Unk | Hill Migrant | Fodder-fuelwood | 0 | Unk |
| ETC_119 | 1 | Male | 55 | 45-64 | Education_Unk | Hill Migrant | Fodder-fuelwood | 0 | Unk |
| ETC_120 | 1 | Female | 39 | 25-44 | Education_Unk | Hill Migrant | Fodder-fuelwood | 0 | Unk |
| ETC_121 | 0 | Female | 38 | 25-44 | Education_Unk | Hill Ethnic | Fodder-fuelwood | 0 | Unk |
| ETC_122 | 1 | Male | 35 | 25-44 | Education_Unk | Dalit | Fodder-fuelwood | 0 | Unk |
| ETC_123 | 1 | Female | 43 | 25-44 | Education_Unk | Indigenous Terai | Fodder-fuelwood | 0 | Unk |
| ETC_124 | 1 | Male | 26 | 25-44 | Education_Unk | Indigenous Terai | Fishing | 0 | Unk |
| ETC_125 | 1 | Male | 57 | 45-64 | Education_Unk | Hill Ethnic | Fodder-fuelwood | 0 | Unk |
| ETC_126 | 0 | Male | 30 | 25-44 | Education_Unk | Hill Ethnic | Fodder-fuelwood | 1 | Unk |
| ETC_127 | 0 | Male | 53 | 45-64 | Education_Unk | Hill Migrant | Livestock herding | 1 | Unk |
| ETC_128 | 0 | Male | 53 | 45-64 | Education_Unk | Hill Migrant | Livestock herding | 1 | Unk |
| ETC_129 | 1 | Male | 39 | 25-44 | Education_Unk | Dalit | Fodder-fuelwood | 0 | Unk |
| ETC_130 | 1 | Male | Unk | Unk | Education_Unk | Indigenous Terai | NTFP colletion | 0 | Unk |
| ETC_131 | 0 | Male | Unk | Unk | Education_Unk | Hill Migrant | Fodder-fuelwood | 1 | Unk |
| ETC_132 | 0 | Male | Unk | Unk | Education_Unk | Hill Migrant | Fodder-fuelwood | 1 | Unk |
| ETC_133 | 0 | Male | Unk | Unk | Education_Unk | Hill Migrant | Fodder-fuelwood | 1 | Unk |
| ETC_134 | 0 | Male | Unk | Unk | Education_Unk | Hill Migrant | Fodder-fuelwood | 1 | Unk |
| ETC_135 | 1 | Female | 37 | 25-44 | Education_Unk | Hill Migrant | Fodder-fuelwood | 0 | Unk |
| ETC_136 | 0 | Female | Unk | Unk | Education_Unk | Hill Ethnic | Fodder-fuelwood | 1 | Unk |
| ETC_137 | 1 | Male | Unk | Unk | Education_Unk | Indigenous Terai | Fishing | 0 | Unk |
| ETC_138 | 1 | Male | Unk | Unk | Education_Unk | Hill Ethnic | Fodder-fuelwood | 0 | Unk |
| ETC_139 | 0 | Male | Unk | Unk | Education_Unk | Indigenous Terai | Fishing | 1 | Unk |
| ETC_140 | 1 | Male | Unk | Unk | Education_Unk | Dalit | Fishing | 0 | Unk |
| ETC_141 | 1 | Male | Unk | Unk | Education_Unk | Hill Ethnic | NTFP colletion | 0 | Unk |
| ETC_142 | 0 | Male | 23 | 15-24 | Education_Unk | Indigenous Terai | Fodder-fuelwood | 1 | Unk |
| ETC_143 | 0 | Male | Unk | Unk | Education_Unk | Hill Migrant | Fodder-fuelwood | 1 | Unk |
| ETC_144 | 0 | Male | 44 | 25-44 | Education_Unk | Hill Migrant | Fishing | 1 | Unk |
| ETC_145 | 1 | Female | 33 | 25-44 | Education_Unk | Hill Migrant | Fodder-fuelwood | 1 | Unk |
| ETC_146 | 1 | Female | 59 | 45-64 | Education_Unk | Hill Migrant | Fodder-fuelwood | 0 | Unk |
| ETC_147 | 0 | Male | 33 | 25-44 | Education_Unk | Indigenous Terai | Fodder-fuelwood | 1 | Unk |
| ETC_148 | 0 | Male | 26 | 25-44 | Education_Unk | Indigenous Terai | Fodder-fuelwood | 1 | Unk |
| ETC_149 | 0 | Male | 60 | 45-64 | Education_Unk | Hill Migrant | Others | 1 | Unk |
| ETC_150 | 0 | Male | 49 | 45-64 | Education_Unk | Hill Ethnic | Fodder-fuelwood | 1 | Unk |
| ETC_151 | 1 | Male | 27 | 25-44 | Education_Unk | Hill Migrant | Fodder-fuelwood | 0 | Unk |
| ETC_152 | 0 | Male | 34 | 25-44 | Education_Unk | Hill Migrant | Fodder-fuelwood | 1 | Unk |
| ETC_153 | 0 | Male | 30 | 25-44 | Education_Unk | Hill Migrant | Fodder-fuelwood | 1 | Unk |
| ETC_154 | 0 | Male | 42 |  | Education_Unk | Hill Migrant | Fodder-fuelwood | 1 | Unk |
| ETC_155 | 1 | Male | 63 | 45-64 | Education_Unk | Indigenous Terai | Fodder-fuelwood | 0 | Unk |
| ETC_156 | 1 | Male | 50 | 45-64 | Education_Unk | Indigenous Terai | Fodder-fuelwood | 0 | Unk |
| ETC_157 | 0 | Female | 58 | 45-64 | Education_Unk | Dalit | Fodder-fuelwood | 0 | Unk |
| ETC_158 | 1 | Male | 63 | 45-64 | Education_Unk | Hill Ethnic | Others | 1 | Unk |
| ETC_159 | 1 | Female | 55 | 45-64 | Education_Unk | Indigenous Terai | Fodder-fuelwood | 1 | Unk |
| ETC_160 | 1 | Female | 45 | 45-64 | Education_Unk | Hill Ethnic | Fodder-fuelwood | 0 | Unk |
| WTC_1 | 0 | Male | Unk | Unk | High school or above | Indigenous Terai | Others | 1 | Upright |
| WTC_2 | 0 | Male | Unk | Unk | Secondary level or below | Indigenous Terai | Others | 1 | Upright |
| WTC_3 | 0 | Female | 28 | 25-44 | Secondary level or below | Hill Migrant | Fodder-fuelwood | 1 | Low lying |
| WTC_4 | 1 | Male | 48 | 45-64 | Secondary level or below | Hill Migrant | NTFP colletion | 1 | Upright |
| WTC_5 | 1 | Female | 67 | 65+ | Secondary level or below | Hill Migrant | NTFP colletion | 0 | Low lying |
| WTC_6 | 0 | Male | Unk | Unk | High school or above | Hill Migrant | Livestock herding | 1 | Upright |
| WTC_7 | 1 | Female | 40 | 25-44 | Secondary level or below | Indigenous Terai | Fodder-fuelwood | 0 | Low lying |
| WTC_8 | 1 | Male | 42 | 25-44 | Secondary level or below | Others | Livestock herding | 0 | Upright |
| WTC_9 | 1 | Female | 32 | 25-44 | Secondary level or below | Indigenous Terai | NTFP colletion | 0 | Low lying |
| WTC_10 | 1 | Female | 65 | 65+ | Secondary level or below | Hill Migrant | Fodder-fuelwood | 0 | Low lying |
| WTC_11 | 1 | Female | 52 | 45-64 | Secondary level or below | Hill Migrant | Fodder-fuelwood | 0 | Low lying |
| WTC_12 | 1 | Female | Unk | Unk | Secondary level or below | Hill Migrant | Fodder-fuelwood | 0 | Low lying |
| WTC_13 | 0 | Male | 36 | 25-44 | Secondary level or below | Others | Fodder-fuelwood | 1 | Upright |
| WTC_14 | 1 | Male | 49 | 45-64 | Secondary level or below | Hill Migrant | Fodder-fuelwood | 0 | Upright |
| WTC_15 | 1 | Female | 48 | 45-64 | Secondary level or below | Hill Migrant | Livestock herding | 1 | Upright |
| WTC_16 | 1 | Male | 45 | 45-64 | Education_Unk | Others | Fodder-fuelwood | 0 | Unk |
| WTC_17 | 0 | Male | 65 | 65+ | Secondary level or below | Indigenous Terai | Livestock herding | 0 | Low lying |
| WTC_18 | 0 | Female | 42 | 25-44 | Secondary level or below | Indigenous Terai | Fodder-fuelwood | 0 | Upright |
| WTC_19 | 1 | Male | 40 | 25-44 | Secondary level or below | Indigenous Terai | Livestock herding | 0 | Upright |
| WTC_20 | 1 | Male | 12 |  | Secondary level or below | Indigenous Terai | Fishing | 0 | Unk |
| WTC_21 | 1 | Male | 31 | 25-44 | Secondary level or below | Indigenous Terai | Fodder-fuelwood | 1 | Low lying |
| WTC_22 | 1 | Male | 42 | 25-44 | Secondary level or below | Indigenous Terai | Fodder-fuelwood | 1 | Unk |
| WTC_23 | 1 | Male | 70 | 65+ | Secondary level or below | Hill Migrant | Livestock herding | 0 | Unk |
| WTC_24 | 1 | Female | 26 | 25-44 | Secondary level or below | Hill Migrant | Fodder-fuelwood | 1 | Low lying |
| WTC_25 | 1 | Male | 71 | 65+ | Education_Unk | Indigenous Terai | Agricultural Work | 0 | Upright |
| WTC_26 | 1 | Male | 61 | 45-64 | Secondary level or below | Indigenous Terai | Livestock herding | 0 | Upright |
| WTC_27 | 1 | Female | 60 | 45-64 | Education_Unk | Hill Migrant | Fodder-fuelwood | 1 | Low lying |
| WTC_28 | 1 | Female | 41 | 25-44 | Secondary level or below | Indigenous Terai | Agricultural Work | 0 | Upright |
| WTC_29 | 1 | Female | 66 | 65+ | Secondary level or below | Hill Migrant | NTFP colletion | 1 | Upright |
| WTC_30 | 1 | Female | 55 | 45-64 | Secondary level or below | Hill Migrant | Fodder-fuelwood | 1 | Low lying |
| WTC_31 | 1 | Female | 49 | 45-64 | Secondary level or below | Hill Ethnic | Fodder-fuelwood | 0 | Upright |
| WTC_32 | 1 | Male | 67 | 65+ | High school or above | Hill Migrant | Fodder-fuelwood | 0 | Low lying |
| WTC_33 | 1 | Male | 27 | 25-44 | Education_Unk | Indigenous Terai | Livestock herding | 0 | Low lying |
| WTC_34 | 1 | Male | 26 | 25-44 | Secondary level or below | Indigenous Terai | HTC response | 1 | Upright |
| WTC_35 | 1 | Female | 49 | 45-64 | Education_Unk | Hill Migrant | Livestock herding | 0 | Upright |
| WTC_36 | 1 | Female | 50 | 45-64 | Education_Unk | Hill Ethnic | Fodder-fuelwood | 1 | Low lying |
| WTC_37 | 1 | Male | 39 | 25-44 | Secondary level or below | Hill Migrant | Fishing | 0 | Upright |
| WTC_38 | 1 | Female | 28 | 25-44 | Secondary level or below | Dalit | Fodder-fuelwood | 1 | Upright |
| WTC_39 | 1 | Male | 65 | 65+ | Secondary level or below | Indigenous Terai | Livestock herding | 0 | Low lying |
| WTC_40 | 1 | Male | 62 | 45-64 | Secondary level or below | Indigenous Terai | Livestock herding | 0 | Upright |
| WTC_41 | 1 | Male | 62 | 45-64 | Secondary level or below | Indigenous Terai | Livestock herding | 0 | Upright |
| WTC_42 | 0 | Male | 40 | 25-44 | High school or above | Hill Migrant | Others | 1 | Upright |
| WTC_43 | 0 | Female | 40 | 25-44 | Education_Unk | Dalit | Fodder-fuelwood | 1 | Low lying |
| WTC_44 | 0 | Male | 51 | 45-64 | Secondary level or below | Dalit | Livestock herding | 0 | Upright |
| WTC_45 | 0 | Male | Unk | Unk | Education_Unk | Dalit | Fodder-fuelwood | 1 | Low lying |
| WTC_46 | 1 | Female | 56 | 45-64 | Secondary level or below | Hill Migrant | Fodder-fuelwood | 1 | Upright |
| WTC_47 | 1 | Male | 62 | 45-64 | Secondary level or below | Indigenous Terai | Livestock herding | 0 | Unk |
| WTC_48 | 1 | Female | Unk | Unk | Education_Unk | Dalit | Fodder-fuelwood | 0 | Unk |
| WTC_49 | 1 | Female | Unk | Unk | Education_Unk | Hill Migrant | Fodder-fuelwood | 0 | Unk |
| WTC_50 | 1 | Male | Unk | Unk | Education_Unk | Hill Migrant | Fodder-fuelwood | 0 | Unk |
| WTC_51 | 1 | Male | Unk | Unk | Education_Unk | Hill Migrant | Fodder-fuelwood | 0 | Unk |
| WTC_52 | 0 | Male | 35 | 45-64 | Education_Unk | Indigenous Terai | Agricultural Work | 0 | Unk |
| WTC_53 | 0 | Male | 46 | 45-64 | Education_Unk | Dalit | Fodder-fuelwood | 1 | Unk |
| WTC_54 | 0 | Male | 65 | 65+ | Education_Unk | Hill Migrant | Livestock herding | 0 | Unk |
| WTC_55 | 0 | Male | Unk | Unk | Education_Unk | Hill Migrant | Fodder-fuelwood | 1 | Unk |
| WTC_56 | 0 | Male | Unk | Unk | Education_Unk | Hill Migrant | Fodder-fuelwood | 1 | Unk |
| WTC_57 | 0 | Male | Unk | Unk | Education_Unk | Hill Migrant | Fodder-fuelwood | 1 | Unk |
| WTC_58 | 0 | Male | Unk | Unk | Education_Unk | Hill Migrant | Fodder-fuelwood | 1 | Unk |
| WTC_59 | 0 | Female | Unk | Unk | Education_Unk | Hill Migrant | Fodder-fuelwood | 1 | Unk |
| WTC_60 | 0 | Male | Unk | Unk | Education_Unk | Hill Migrant | Fodder-fuelwood | 1 | Unk |
| WTC_61 | 0 | Female | Unk | Unk | Education_Unk | Hill Ethnic | Fodder-fuelwood | 1 | Unk |
| WTC_62 | 0 | Male | Unk | Unk | Education_Unk | Hill Migrant | Fodder-fuelwood | 1 | Unk |
| WTC_63 | 0 | Male | Unk | Unk | Education_Unk | Indigenous Terai | Fodder-fuelwood | 1 | Unk |
| WTC_64 | 0 | Male | Unk | Unk | Education_Unk | Indigenous Terai | Fodder-fuelwood | 1 | Unk |
| WTC_65 | 0 | Male | Unk | Unk | Education_Unk | Indigenous Terai | Fodder-fuelwood | 1 | Unk |
| WTC_66 | 0 | Male | Unk | Unk | Education_Unk | Indigenous Terai | Fodder-fuelwood | 1 | Unk |
| WTC_67 | 0 | Male | Unk | Unk | Education_Unk | Indigenous Terai | Fodder-fuelwood | 1 | Unk |
| WTC_68 | 1 | Male | 25 | 25-44 | Education_Unk | Hill Migrant | Fodder-fuelwood | 0 | Unk |
| WTC_69 | 0 | Male | 28 | 25-44 | Education_Unk | Dalit | Agricultural Work | 1 | Unk |
| WTC_70 | 1 | Female | 45 | 45-64 | Education_Unk | Hill Migrant | Fodder-fuelwood | 0 | Unk |
| WTC_71 | 0 | Male | 28 | 25-44 | Education_Unk | Hill Migrant | Fodder-fuelwood | 1 | Unk |
| WTC_72 | 1 | Female | 25 | 25-44 | Education_Unk | Dalit | Fodder-fuelwood | 0 | Unk |
| WTC_73 | 1 | Female | 41 | 25-44 | Education_Unk | Indigenous Terai | Agricultural Work | 0 | Unk |
| WTC_74 | 1 | Female | 28 | 25-44 | Education_Unk | Hill Migrant | Livestock herding | 0 | Unk |
| WTC_75 | 0 | Female | 45 | 45-64 | Education_Unk | Hill Migrant | NTFP colletion | 1 | Unk |
| WTC_76 | 0 | Female | 53 | 45-64 | Education_Unk | Hill Migrant | NTFP colletion | 1 | Unk |
| WTC_77 | 0 | Female | 18 | 15-24 | Education_Unk | Hill Migrant | NTFP colletion | 1 | Unk |
| WTC_78 | 1 | Female | 52 | 45-64 | Education_Unk | Hill Migrant | Fodder-fuelwood | 0 | Unk |
| WTC_79 | 1 | Female | 35 | 25-44 | Education_Unk | Hill Migrant | Fodder-fuelwood | 1 | Unk |
| WTC_80 | 1 | Male | 71 | 65+ | Education_Unk | Hill Migrant | Others | 1 | Unk |
| WTC_81 | 0 | Male | 30 | 25-44 | Education_Unk | Hill Migrant | Others | 1 | Unk |
| WTC_82 | 1 | Female | 23 | 15-24 | Education_Unk | Hill Migrant | Agricultural Work | 0 | Unk |
| WTC_83 | 1 | Female | 32 | 25-44 | Education_Unk | Dalit | Fodder-fuelwood | 0 | Unk |
| WTC_84 | 1 | Female | Unk | Unk | Education_Unk | Hill Migrant | Fodder-fuelwood | 0 | Unk |
| WTC_85 | 0 | Female | 30 | 25-44 | Education_Unk | Indigenous Terai | Fodder-fuelwood | 1 | Unk |
