# Supplementary material for: Tigers in the Neighborhood: Factors Associated With Fatal Tiger Attacks in a Human‐Dominated Landscape, Nepal
Source: Ecol Evol. 2026 Aug 16;16(8):e74206. doi: 10.1002/ece3.74206 (PMC13478372; doi:10.1002/ece3.74206)
Supplement: Supplementary file 3 [file ECE3-16-e74206-s003.docx]

**Tigers in the neighborhood: Factors associated with fatal tiger attacks in a human-dominated landscape, Nepal**

Bed Kumar Dhakal, Babu Ram Lamichhane, Sanjay Kandel, Naresh Subedi, Matthew Low, Thakur Silwal

Supplementary File S2. R Code for modelling fatality risk on tiger attack.

library(lme4)

library(MuMIn)

#Set working directory and read the data

HTC<-read.csv("Risk of fatality.csv", header= TRUE)

head(HTC)

summary(HTC)

#Tiger GLM

HTC_B <- glm(formula=Death_injury ~ Group+Sex_victim+Age_category+Education_Victim+Ethnicity_victim+Activity,

data=HTC, family=binomial)

out.tig<-step(HTC_B, trace=0)

summary(out.tig)

#Tiger GLM interaction

HTC_B1<-glm(formula=Death_injury ~ Group+Sex_victim+Education_Victim+Activity+Posture+Activity+Sex_victim*Activity+Sex_victim*Education_Victim,

data=HTC, family=binomial, na.action="na.fail")

#multimodel inference

#remove missing data

Tig<-HTC[complete.cases(HTC),]

head(Tig)

#rerun model

ML1<-glm(formula=Death_injury ~ Group+Sex_victim+Age_category+Education_Victim+Ethnicity_victim+Activity+Posture,

data=HTC, family=binomial, na.action="na.fail")

summary(ML1)

ML2<-glm(formula=Death_injury ~ Group+Sex_victim+Age_category+Education_Victim+Ethnicity_victim+Activity+Posture+Sex_victim*Posture+Sex_victim*Age_category+Sex_victim*Activity+Group*Posture,

data=HTC, family=binomial, na.action="na.fail")

summary(ML2)

ML3<-glm(formula=Death_injury ~ Sex_victim+Age_category+Education_Victim+Ethnicity_victim+Activity,

data=HTC, family=binomial, na.action="na.fail")

summary(ML3)

ML4<-glm(formula=Death_injury ~ Group+Sex_victim+Age_category+Education_Victim+Ethnicity_victim+Activity+Posture+Sex_victim*Posture+Sex_victim*Age_category+Sex_victim*Activity+Group*Posture,

data=HTC, family=binomial, na.action="na.fail")

drop1(ML2, test="LRT")

write.table(drop1(ML2, test="LRT"), file = "LRT_Test.csv", append = TRUE, sep = ",")

drop1(ML4, test="LRT")

write.table (drop1(ML4, test="LRT"), file = "LRT_Test.csv", append = TRUE, sep = ",")

#this take some time

ML_dredge<-dredge(ML1)

head(ML_dredge)

ML_dredge2<-dredge(ML2)

ML_dredge3<-dredge(ML3)

write.table(ML_dredge2, file="mod_sel.csv", sep = ",")

ML_dredge4<-dredge(ML4)

subset (ML_dredge4, delta=10)

head(ML_dredge2)

#view the top model

subset(ML_dredge, delta==0)

subset(ML_dredge3, delta==5)

sim<-subset(ML_dredge, delta<2)

sim[1,]

importance(sim)

sim2<-subset(ML_dredge3, delta<10)

sim4<-subset(ML_dredge4, delta<5)

sim4

write.table(sim4, file="mod_sel.csv", sep = ",")

summary(sim2)

sim2[1,]

sw(sim2)

#Final model

HTC_fin<-glm(formula=Death_injury ~ Group+Sex_victim+Education_Victim+Posture,

data=Tig, family=binomial, na.action="na.fail")

summary (HTC_fin)

write.table(summary(HTC_fin), file="mod_out1.csv", sep = ",")

#mode average over models less than 2 AICc unit difference

MA.ests<-model.avg(ML_dredge, subset= delta < 5, revised.var = TRUE)

summary(MA.ests)

MA.ests$coefficients

MA.ests$importance

importance(MA.ests)

plot(MA.ests$coefficients)
