# Supplementary material for: Tigers in the Neighborhood: Factors Associated With Fatal Tiger Attacks in a Human‐Dominated Landscape, Nepal
Source: Ecol Evol. 2026 Aug 16;16(8):e74206. doi: 10.1002/ece3.74206 (PMC13478372; doi:10.1002/ece3.74206)
Supplement: Supplementary file 4 [file ECE3-16-e74206-s004.docx]

**Tigers in the neighborhood: Factors associated with fatal tiger attacks in a human-dominated landscape, Nepal**

Bed Kumar Dhakal, Babu Ram Lamichhane, Sanjay Kandel, Naresh Subedi, Matthew Low, Thakur Silwal

**Supplementary File S4. Synthesis of victims' and family members' perspectives on causes of tiger movement near settlements and measures for reducing tiger attacks, Nepal Terai Arc Landscape.**

*Based on open-ended interview prompts (n = 122 respondents for 'Reason tigers come near settlements' and n = 144 for 'Measures for saving humans from tiger attacks'; non-response 50% and 41%, respectively). Percentages are calculated within respondents who answered each question (column denominator). Cross-tabulations by management complex (ETC = Eastern Terai Complex, comprising Chitwan–Parsa; WTC = Western Terai Complex, comprising Banke–Bardia–Shuklaphanta) and by victim sex are shown for descriptive purposes only.*

**Section A. Reason tigers come to settlements (n = 122)**

**Supplementary File S4 (a). Respondent-perceived reasons for tiger movement into settlements**

| **Respondent-perceived reason** | **Overall n = 122** | **ETC n = 85** | **WTC n = 37** | **Male n = 82** | **Female n = 40** |
| --- | --- | --- | --- | --- | --- |
| Movement in search of prey | 82 (67.2) | 46 (54.1) | 36 (97.3) | 52 (63.4) | 30 (75.0) |
| Presence of an individual conflict-causing/problematic tiger | 15 (12.3) | 15 (17.6) | 0 | 12 (14.6) | 3 |
| Lack of fencing at the forest–settlement interface | 7 (5.7) | 7 (8.2) | 0 | 6 (7.3) | 1 |
| Settlement located immediately adjacent to dense forest | 6 (4.9) | 6 (7.1) | 0 | 5 (6.1) | 1 |
| Incidental tiger movement into settlements | 6 (4.9) | 6 (7.1) | 0 | 4 | 2 |
| Increase in tiger population | 3 | 2 | 1 | 1 | 2 |
| Habitat loss/degradation | 3 | 3 | 0 | 2 | 1 |
| **Total respondents answering** | **122** | **85** | **37** | **82** | **40** |

**Note.** Values are counts and percentages within each respondent stratum. Percentages are shown only for cells with ≥5 respondents.

*******The most frequently reported perceived reason for tiger movement near settlements was prey-seeking behaviour, identified by 82 of 122 respondents (67.2%). This perception was especially dominant among WTC respondents, where 36 of 37 respondents (97.3%) attributed tiger entry into settlements to prey searching, compared with 46 of 85 ETC respondents (54.1%). Other perceived reasons were less frequently reported and included the presence of an habituated conflict-causing tiger, lack of fencing or protective barriers at the forest–settlement interface, settlement proximity to dense forest, incidental movement, tiger population increase, and forest disturbance or habitat loss.******

**Supplementary File S4 (b). Measures for saving humans from tiger attack (n = 144).**

| Intervention level | Theme | Overall | ETC | WTC | Male | Female |
| --- | --- | --- | --- | --- | --- | --- |
|  | *(respondents answering, n)* | *144* | *100* | *44* | *97* | *47* |
| Behavioural risk reduction | Safer collective forest use; avoidance of high-risk areas and times | 125 (87%) | 87 (87%) | 38 (86%) | 85 (88%) | 40 (85%) |
| Protective infrastructure | Fencing and safer access routes | 16 (11%) | 11 (11%) | 5 (11%) | 10 (10%) | 6 (13%) |
| Institutional response | Targeted management of habitually conflict-causing tigers | 1 | 1 | 0 | 1 | 0 |
| Ecological management | Habitat and prey-base management | 2 | 1 | 1 | 1 | 1 |

*Notes: Responses were elicited through open-ended prompts and coded into the themes shown here by trained interviewers during questionnaire administration. Multiple-response coding was not permitted; respondents selected the single most salient reason or measure. Non-response was high (≈50% for the reasons item and ≈41% for the measures item).*

*****Behavioural risk-reduction measures were the dominant respondent-proposed strategy for reducing the risk of tiger attacks on people, reported by 125 of 144 respondents (86.8%). This pattern was consistent across management complex and respondent sex, with similar proportions among ETC respondents (87.0%), WTC respondents (86.4%), male respondents (87.6%), and female respondents (85.1%). Protective infrastructure was the second most frequently reported measure, whereas institutional response and ecological management were rarely mentioned.****
